# Deferring draft picks: Empirical analysis of the AFL draft

**DOI:** 10.1371/journal.pone.0311240

**Published:** 2024-09-27

**Authors:** Jemuel Chandrakumaran, Paul Larkin, Sam McIntosh, Sam Robertson

**Affiliations:** 1 Institute for Health and Sport (IHES), Victoria University, Melbourne, Australia; 2 Maribyrnong Sports Academy, Melbourne, Australia; 3 Western Bulldogs Football Club, Melbourne, Australia; La Trobe University, AUSTRALIA

## Abstract

Trading picks is a common transaction observed in sporting leagues that employ player drafts. However, many current and revised draft formats employed by professional leagues allow for the trading of picks in the current year for those in the future. This in turn raises a question of an appropriate rate of discount which can be used to account for the delay in relinquishing current picks for those in the future. This study proposes a discount rate function in the Australian Football League based on the difference between a player’s contribution to his team in his first season to that of his career. The findings reveal a varying discount rate that decreases with the progression of the draft ranging between 42% and 51%, given the increasing deviation of post draft outcomes in the same range. Furthermore, after discounting said picks for four years, the variance in pick reduced considerably as poor performers are delisted after their initial contract period. Upon adopting the model to evaluate previous trades, it is evident that almost all parties infer a higher value on current year selections than future selections (discounting future picks by up to 120%), seeking instant returns over deferred options. Hence, a decision maker trying to capitalise on pick value only would be better off deferring the right to choose in the current year.

## Introduction

Player drafts are commonly used in closed sports leagues as a way to disperse amateur talent in an equitable manner. Introduced first in the National Football League (NFL), the draft used a reverse order system based on each team’s season standing [1]. For example, the team finishing last immediately before the draft would have the first pick followed by the second last team and so on. Once a complete set of selections have been made, the process is repeated again (around seven times in the current NFL draft). Owing to its success, derivatives of the draft have been incorporated in various other leagues including the National Hockey League (NHL), National Basketball Association (NBA), and Major League Baseball (MLB). In order for teams to pursue their own interests in terms of recruitment, leagues have allowed for the trading of picks, essentially making the selections themselves financial assets owned by the benefitting clubs. When prompted, a team can either opt to exercise the pick and choose an amateur player to strengthen its list; or trade the pick for a player(s) and/or pick(s). As the assets in question are both indivisible and come from different classes, ample guidance has been given in the prevailing literature to facilitate the value-in-exchange similar to a barter system [2, 3].

In addition to this, leagues have also allowed for the trading of current year picks for those in the future [4]. Whilst unlike the previous issue of comparing different asset classes, these exchanges will require the evaluation of picks across different time periods. Any set of intertemporal choices happening across different time periods, should be discounted whereby the utility of consumption should be perceived at the present time [5]. In a purely financial sense, investment decisions involving multi-year payoffs are discounted based on the cost of capital. For example, a mutual fund scheme returning $2 at the end of year with an initial investment of $1 today, cannot be automatically attributed a profit of $1 ($2-$1). As the profit is realised in a year’s time, this should be discounted to today’s terms by the cost of capital. Assuming this is 10%, the $2 is worth $1.82 today (2/1.1). Hence, the true profit of this scheme would be $0.82 instead. Yet, when such decisions involve a non-monetary return, an implied discount rate is used to assess the perceived difference between current and future payoffs. However, these implied rates generally include other psychological factors on top of the discounted utility aspect. The theory of intertemporal consumption suggests that an individual’s preferences in terms of consumption or saving varies across their lifetime. Early attempts to refine this theory showed shifts in behaviour where individuals would prioritise saving over spending in their middle years in effort to secure their retirement [6]. Traditional models of economics assumes that any rational agent’s preference would decrease monotonically with increased time delay [7]. The basic idea here is that when all things remain equal, an agent would prefer to have something now as opposed to later [8].

Within the context of the draft, let’s assume team A is eyeing a key amateur in the current year’s drafting cohort and has only pick nine in the current year. This team would then have to agree with team B to exchange picks from their future years to obtain a current year pick, earlier than their spot at nine (let’s say pick five). However, team B would not value picks in the future to be of equivalent standing to those in the current year as there is a delay in realising the benefit of those picks. Hence team B would evaluate any proposal from team A to relieve its fifth pick in the current year by discounting team A’s picks from the future. Previously, Massey and Thaler [9] estimated an implied discount rate of 136% for multi-year trades by managers in the NFL by equating each trade over two decades using a Weibull distribution. That is, if pick 5 is worth 100 today, the same pick from next year’s draft has been estimated to be worth 64 by NFL decision makers (100*(100–36)). Taylor et al. [10] found that this rate ranged between -172% and 626%, owing to the need of clubs favouring current year picks (and by extension, access to high-quality recruits early on) to advance their goal of building a winning roster. Needless to say, these competing interests create arbitrage opportunities for the parties involved in the trade to profit off the other, causing an inefficient trading market. These implied discount rates reflect market rates and include both the actual discount factor and any gain/loss asymmetry, such as a team’s individual requirements to hold a list spot open or tackling the unknown of what the future draft pick position might be, which teams attribute on top. This still leaves the question, of the manner in which future picks should be discounted. Hence, through this paper we aim to introduce a discount function based on historic player performance data (unlike Massey and Thaler [9], who looked at historical trade data) which can used to evaluate pick trades involving current and future year draft picks.

## Empirical analysis of the AFL draft

In order to answer the question of an appropriate discount rate, we used the AFL national player draft due to its uniqueness and effectiveness in the trade market. Unlike most North American leagues, the AFL conducts a smaller draft and requires a team to maintain a smaller roster, indirectly promoting sequential selection parity, where the performance of players selected through the draft closely resembles the pick used to recruit them [11]. Furthermore, as each team has to have 18 players on field per game, the impact of a single player (and/or position) in a game is significantly less when compared to other sporting codes like the NBA or NHL, making the talent pool more homogenous. Incorporated in 1986, the AFL national player draft is a labour market intervention technique used to discourage expensive bidding wars and equitably distribute amateur talent [12]. It uses a reverse order system, allowing the team that finished last in the season immediately prior to the draft to select first, and so on. After all teams complete a set (currently 18; referring to the number of teams in the AFL) of selections (known as a round), the process is repeated until each team has made enough selections to fit within the list size guidelines. This is typically around four-to-five rounds in the current format of the draft. Through this, the league hopes to cyclically alter the fortunes of all teams and deny the continued dominance of a select few [13], assuming teams choose the best (optimal choice [14]) player with each pick. Similar to its North American counterparts the AFL draft allows teams to trade picks for active players, and other picks. The current body of knowledge facilitates the evaluation of such exchanges [15–17], except those involving picks from the future.

### Data

In order to create the discount rate models, data on all draftees selected between 2003 and 2016 together with their performance from 2004 to 2017 was collected from Sorenson Technologies and supplemented using third-party websites (including footywire.com, afltables.com, and draftguru.com). This yielded a sample of 1,123 players. As the AFL allows teams to draft players who have played in the league before and elevate amateurs from their rookie list to the senior squad through the draft, such observations were removed from the sample to avoid any biases they might infer when compared to their amateur counterparts. Furthermore, to have a consistent endpoint, draftees selected after pick 73 (this is also the end point of the league-administered pick value function [18]–though a future iteration of this in 2025 would stop at pick 53 [19]) were eliminated, which coincidentally included a majority of the observations filtered in the previous exception. This left 905 career cross-sections and 4,781 player seasons. Table 1 describes the primary variables used throughout this study.

**Table 1:** Description of the independent and dependent variables used in the models.

Variable	Description
**Dependent Variables**	
Games Played	The number of regular season games played throughout a draftee’s career. Post-season matches are not included as it may create an inherent bias towards players from successful teams who get to play more games.
Brownlow Medal Votes (BMV)	BMV are awarded by umpires to determine the league’s best and fairest. The top three players for each game are awarded these votes, with the best player getting three votes, whilst the second and third get two and one respectively. This variable accounts for the total BMV accumulated throughout their career in the above games.
Contribution to Margin of Victory (CMV)	The CMV metric was obtained by regressing the difference of a set of on-field metrics such as kicks and marks, which translated to point differences in a regular season AFL game between 2003 and 2017 as shown in S1 Table. After refining the predictors using a stepwise analysis accounting for Bayesian Information Criterion, the coefficients in model 9 of S1 Table were used to extrapolate the career and seasonal CMV per draftee used in this study. This variable has been used in similar studies [11, 20] and mimics the process followed in other similar works [21–23], including the proprietary Champion Data player ranking points that is widely used in the AFL.
**Independent Variables**	
Drafting Age	Age of the draftee in the year when he was drafted.
Indigenous	Indicator variable to identify if a draftee is Indigenous.
Father–son (F/S)	This is a special selection type whereby teams select the sons of former players who have played 100 games for the respective club. Within the sample time period, the rule was exercised under three different regimes. Given the nature of these changes, it was essential to capture these effects (if any) similar to previous works [11, 24]. ≤2006 = Team that chose F/S players in or prior to the 2006 draft were only meant to compensate the competing bid for the same pick with a third-round pick. Due to this, the expected performance of such players did not reconcile with the pick used to select them. 2007 ≤ 2014 = For F/S players chosen between 2007 and 2014 teams were meant to match a bid for the same player with a pick in the same round. While this is said to have reduced the mismatch, there was still a chance for a material difference in outcomes. ≥2015 = The DVI was introduced in 2015, whereby teams choosing F/S players had to compensate competing bids with equivalent draft points.
Club academy (C/A)	Similar to F/S players, this is also another special category whereby teams recruit players from their own junior academies to join their senior ranks. Teams from New South Wales and Queensland were given the opportunity to develop amateur Australian Football talent through these academies, in order to make them ready for potential careers in the AFL, as young athletes in these regions usually followed the premier rugby league competitions instead due to its overwhelming popularity. It also follows the same rules as F/S in terms of draft selection. Since it was introduced only in 2011, they only follow the last two iterations of the F/S rule. ≤ 2014 = For C/A players chosen up to 2014 teams were meant to match a bid for the same player with a pick in the same round. ≥2015 = The DVI was introduced in 2015, whereby teams choosing C/A
Height	Height in centimetres.
Weight	Weight in kilograms.
Position	The most common position played by the player in their career.
Drafting Team	Team that drafted the player.
Amateur League	The amateur league from which the draftee is recruited.

**Fig 1:**
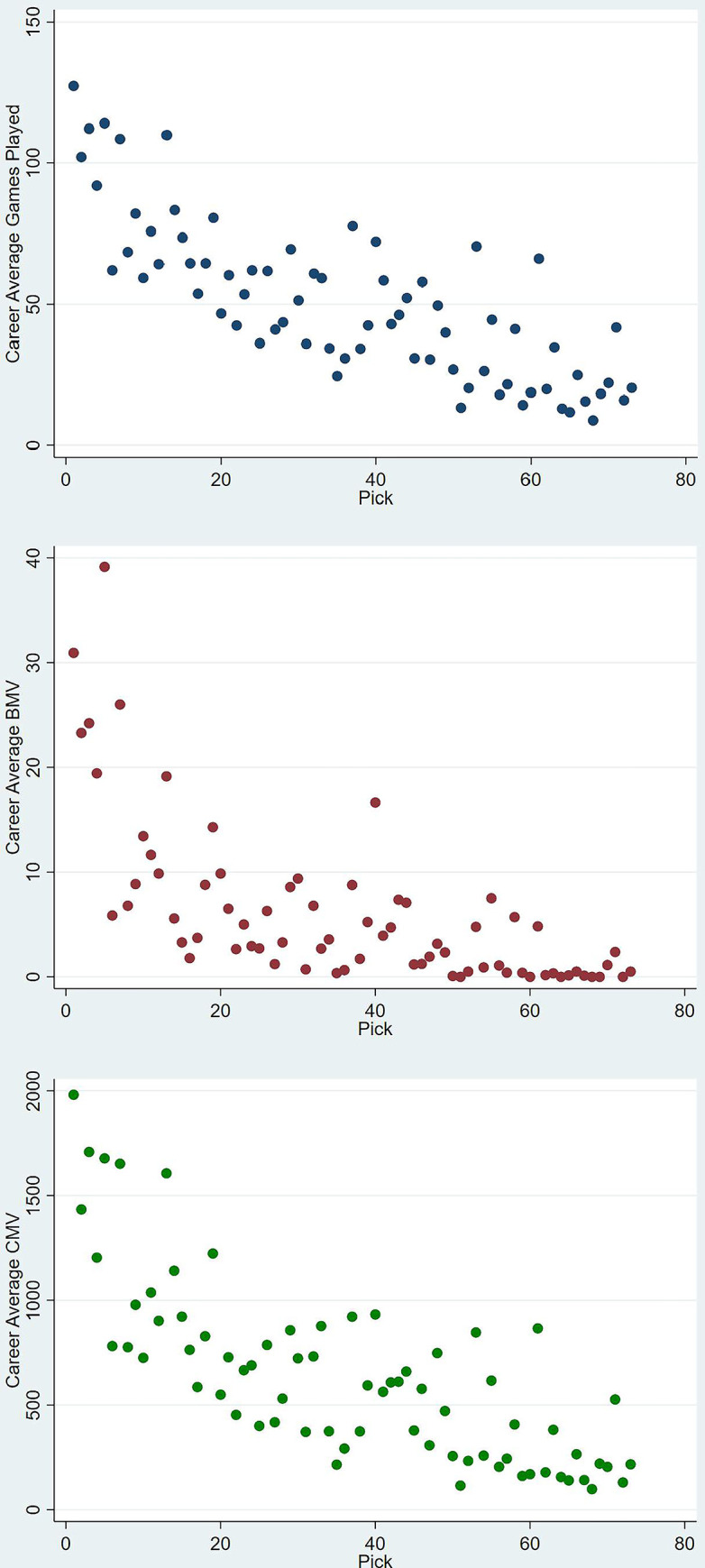
Dispersion of career performance metrics grouped based on draft picks.

**Fig 2:**
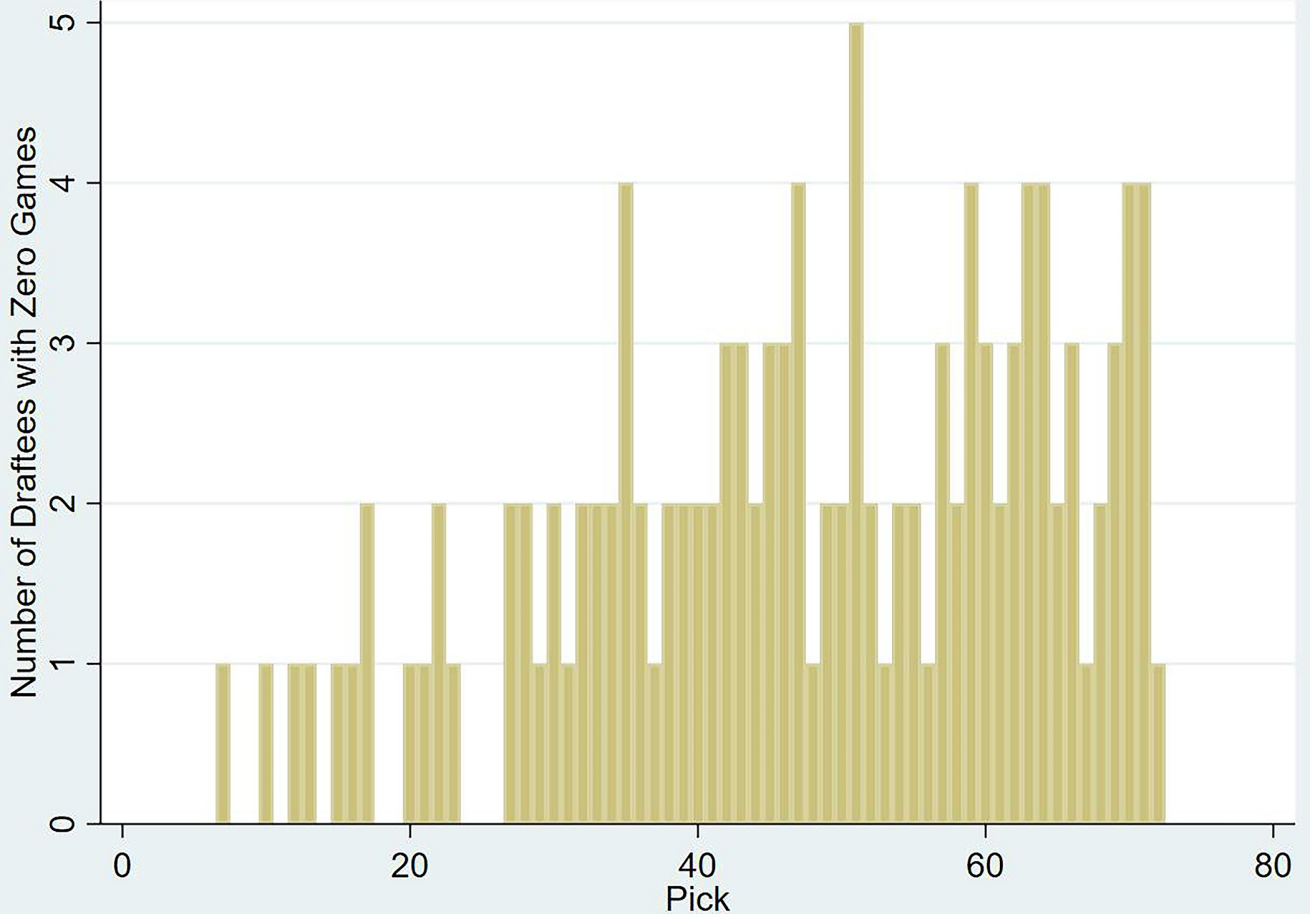
Dispersion of draftees who did not play in the AFL and their associated draft pick.

The descriptive statistics of all continuous variables used are presented in Table 2. On average, draftees play 54 games in their career, earning 7 Brownlow Medal Votes (BMV), and contributing 680 points to their team’s victory margin (CMV, a weighted sum of in-game statistics with the weights derived by a linear regression in model 9 of S1 Table). The distribution of these dependent performance variables (games played, BMV, and CMV) is shown in S1–S3 Figs together with their averages per draft pick in Fig 1. On the other hand, and consistent with previous findings, there was minimal deviation in terms of the personal metrics such as drafting age, height, and weight [25]. Yet, upon viewing the dispersion of draftees who have not played at least one game in the league, the density function in Fig 2 shows a growing trend with the progression of the draft that drops in the tail, mimicking the performance risk associated with such selections.

**Table 2:** Descriptive statistics of continuous variables used in the models.

Variable	Mean	Median	Min	Max	Inter-Quartile Range	Standard Deviation
Games Played	53.56	28.00	0.00	260.00	81.00	60.75
BMV	6.73	0.00	0.00	181.00	3.00	19.49
CMV	680.24	264.57	0.00	5,011.71	957.36	933.55
Drafting Age	18.16	18.00	17.00	26.00	0.00	1.07
Weight	84.93	84.00	63.00	112.31	10.39	7.86
Height	188.14	187.65	167.81	208.88	10.00	6.84

Upon reviewing the listing statistics in Table 3, almost all draftees were listed in the first two seasons, in line with the length of their initial player contracts [26]. However, the conversion of draftees who actually played, against those who were listed, trends above 80% from the third season onwards, upon the expiry of the initial contract, as teams only keep those with promising prospects within their ranks [27]. This is further reflected by the rise in the performance metrics from the third season onwards (games played, BMV, CMV). However, when reviewing the performance (in terms of CMV) within the first three seasons, each year a player’s performance increases by a factor of their first season, further warranting the need for this study to quantify the delay in materialising the benefit of drafting a player. This delay is thereby estimated using two models in the following sections.

**Table 3:** Career listing outcomes of draftees and their respective performance since being drafted.

Years Since First Drafted	No. of Draftees Eligible to Play	No. of Draftees Listed in the year	No. of Draftees who Played in the year	Averages per year of listed draftees					Year-on-Year CMV increase
				Age	Pick	Games Played	BMV	CMV	
1	905	905	531	19.17	33.69	4.91	0.15	48.23	-
2	833	828	610	20.14	33.30	7.73	0.38	84.58	1.75
3	766	686	575	21.12	31.48	9.84	0.80	117.35	1.39
4	697	570	504	22.09	30.03	11.77	1.53	149.98	1.28
5	638	447	408	23.05	28.34	12.59	1.76	166.90	1.11
6	578	371	335	24.03	28.06	13.18	2.19	181.31	1.09
7	513	289	267	24.99	26.83	13.74	2.28	191.87	1.06
8	445	226	213	25.94	26.09	14.06	2.73	197.97	1.03
9	382	172	160	26.92	25.08	14.65	3.04	209.51	1.06
10	312	126	121	27.92	25.32	14.53	3.34	210.48	1.00
11	249	87	80	28.85	26.38	13.97	2.55	196.96	0.94
12	179	45	39	29.62	21.69	13.16	3.09	206.28	1.05
13	118	23	21	30.57	21.91	12.39	1.78	171.07	0.83
14	57	6	6	31.33	33.67	10.83	0.83	161.86	0.95

Based on the differing value inferred per pick by teams, it is reasonable to hypothesize that any proposed discount rate should also vary per pick as the expectations in terms of performance differs with the progression of the draft. Moreover, as the actual performance of draftees deviates considerably amongst later selections (as referenced in Fig 2 and in Tuck and Richards [28], where the difference between the fifth percentile and median performance per pick grew with higher picks), it is also reasonable to assume that this discount rate would decrease as the risk is already factored in to the value of the pick, creating the basis for the models discussed hereon.

### First season as a percentage of overall career performance

This section uses a three-step procedure to obtain an estimate of a discount function. First, define a new dependent variable which can used a proxy to quantify the delay. Next, an empirical model is specified to find the determinants (other than draft pick number) of player performance. The third step involves fitting the relationship estimated previously with the associated draft picks to create the discount function.

Before defining the parameters for this model, the dependent performance metrics were repurposed to calculate a draftee’s percentage contribution in their first season as a product of their career. That is, if a draftee played 10 games in their first season and 100 games in their career, the new dependant variable would attribute a value of 10% (the same was done for the other metrics, BMV and CMV). Similarly, if a draftee had not yet played a game within the sample range, the dependent variable would be 0.

A linear regression model with fixed effects for the drafting year was estimated (bounded generalized linear models yielded similar outcomes) using the independent variables outlined in Table 1 for each of the three-performance metrics (derivatives of the dependent variables). The sample was further curtailed to only include draftees who could have played more than two seasons (as a draftee’s contribution to their team usually begins from their third season [29], i.e., draftees selected between 2003 and 2014). This allowed us to negate the effects of draftees still in their initial contracts as shown in Table 3. The age when drafted, height, and weight together with indicator variables for race, position played, drafting team, and amateur league were used as the determinants (in line with Chandrakumaran [11] and Stewart et al. [24]). Furthermore, indicators for draftees selected using specialist picks (i.e., Father-Son (F/S) and Club-Academy (C/A)) were included to the list of determinants broken up based on the rule changes within the sample period (refer to Table 1).

The results shown in Table 4 suggest that drafting age had a positive effect, as older recruits tend to have shorter careers than their younger counterparts. This in turn requires them to have more game time in their first season as their careers might be relatively smaller. Furthermore, draftees selected by Carlton played 5% of their career in the first season. However, Gold Coast draftees reached up to 10%. This could be due to the fact that Gold Coast entered the league only in 2011, giving its players a shorter life span within the sample evaluated in this study. Furthermore, Gold Coast could have also utilised most of their new recruits to understand their potential as they might not have had veteran players within the squads at this time. C/A players recruited prior to 2015 showed similar results, which could be attributed to the same effect observed with Gold Coast as C/A was first introduced in 2011. The indigenous player indicator returned insignificant values in all models failing to materialise the performance anomalies observed in previous works in terms of game time [30] and BMV [31]. The models that were run as a check for robustness excluding those who did not play returned similar outcomes as the dispersion of players who did not play a game, though positively skewed, was evenly spread as shown in Fig 2.

**Table 4:** First season performance as a percentage of career model.

Model Type	Linear Regression with Fixed Effects for Year Drafted					
Dependant Variable	First Season as a Percentage of Career					
	Games Played		BMV		CMV	
Model Number	1		2		3	
Independent Variables	Coefficient	Robust Std. Err.	Coefficient	Robust Std. Err.	Coefficient	Robust Std. Err.
Drafting Age	0.03***	0.01	0.02*	0.01	0.03***	0.01
Club academy (C/A)						
≤ 2014	0.19***	0.03	0.04**	0.02	0.18***	0.06
Father–son (F/S)						
≤ 2006	0.03**	0.01	-0.01	0.01	0.04*	0.02
2007 ≤ 2014	-0.01	0.04	-0.03	0.01	-0.03	0.03
Indigenous	0.06*	0.03	0.01	0.02	0.05*	0.03
Weight	-0.01***	0.01	0.09	0.01	-0.01***	0.01
Height	0.01*	0.01	-0.01	0.01	0.01	0.01
Position Played ^a^						
Forward	0.04*	0.02	-0.02	0.01	0.04*	0.02
Midfielder	0.01	0.01	0.01	0.01	-0.01	0.02
Ruckman	0.02	0.02	-0.02	0.02	0.03	0.02
Drafting Team ^b^						
Brisbane	0.03	0.05	0.04	0.02	0.04	0.04
Carlton	0.06**	0.02	0.03	0.02	0.06***	0.02
Collingwood	0.05	0.04	0.05	0.03	0.05	0.04
Essendon	0.03	0.03	0.02	0.01	0.01	0.03
Fremantle	0.06	0.05	0.08	0.05	0.08	0.06
Geelong	-0.01	0.04	0.01	0.01	0.01	0.03
Gold Coast	0.10**	0.04	0.02*	0.01	0.11**	0.04
Greater Western Sydney	0.01	0.04	0.04*	0.02	-0.01	0.04
Hawthorn	-0.01	0.04	0.01	0.01	-0.01	0.04
Melbourne	0.05	0.04	0.04	0.02	0.04	0.04
North Melbourne	0.01	0.03	0.01	0.01	-0.01	0.02
Port Adelaide	0.01	0.03	0.01	0.01	0.01	0.02
Richmond	0.07*	0.03	0.03	0.02	0.06	0.04
St. Kilda	0.07	0.07	0.04	0.02	0.05	0.07
Sydney	-0.02	0.03	0.06*	0.03	-0.02	0.03
Westcoast	0.07	0.05	0.04	0.02	0.08	0.04
Western Bulldogs	0.03	0.05	0.02**	0.01	0.04	0.05
Amateur League ^c^						
SANFL	0.01	0.03	0.05*	0.02	0.02	0.03
TAC Cup	0.03	0.03	0.03*	0.01	0.04	0.03
WAFL	0.01	0.02	0.04*	0.01	0.01	0.02
Constant	-0.52**	0.18	-0.39	0.24	-0.56**	0.19
Observations	689		689		689	
Listed Players Drafted Between	2003–14		2003–14		2003–14	
R-squared	0.16		0.06		0.15	
Prob(F-Statistic)	0.00		0.00		0.00	

^a^ the reference position is Defender

^b^ the reference team is Adelaide Crows

^c^ the reference league is Other.

*** p<0.01

** p<0.05

* p<0.1

**Fig 3:**
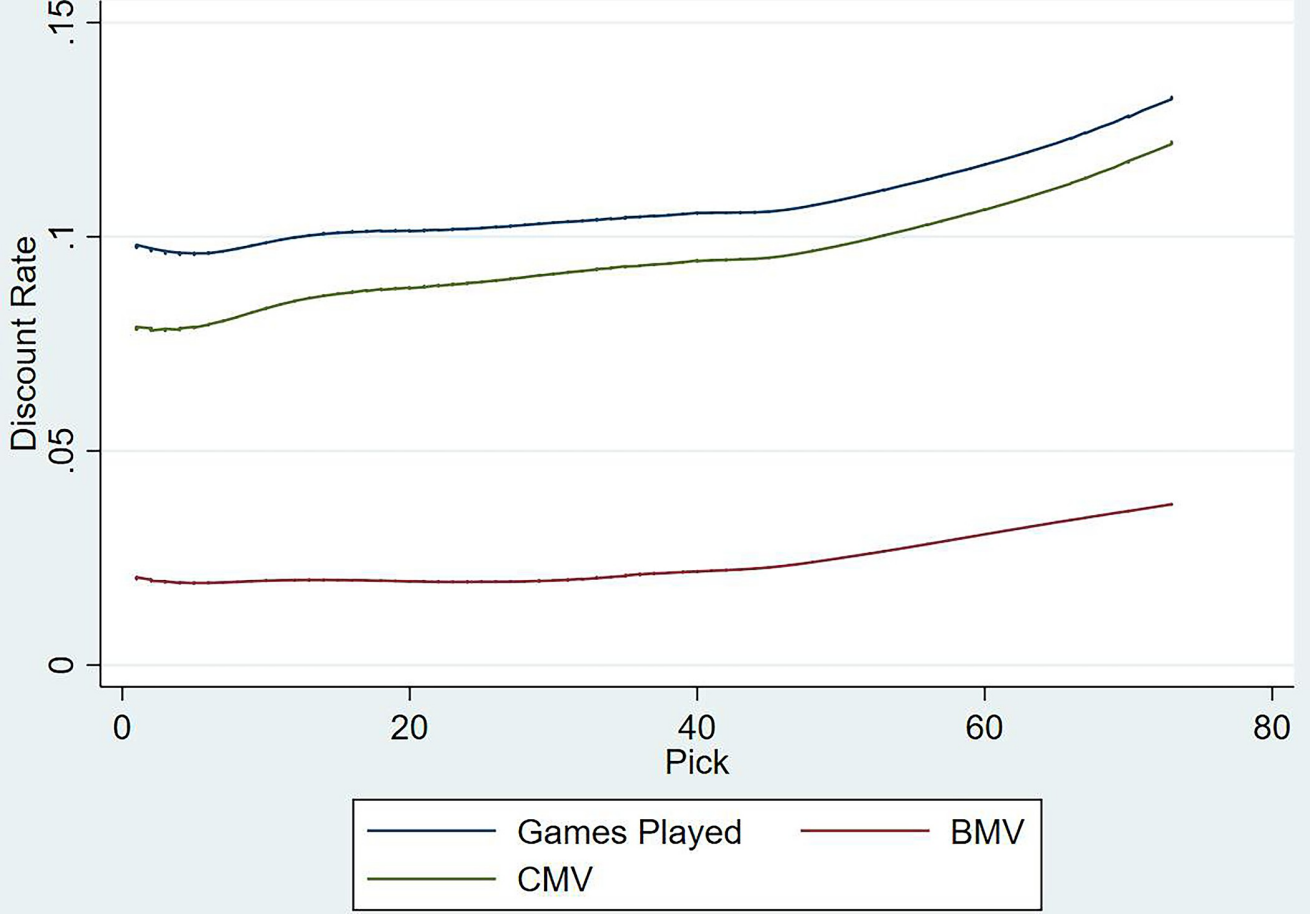
Predicted discount rate as outlined by the first season performance of a draftee as a percentage of career performance per pick.

In order to the create the discount function, the expected outcomes of the previous model for all three-performance metrics were then fitted against pick number using a locally weighted scatterplot smoothing (LOWESS) curve, as shown in Fig 3 (the fitted plots including confidence intervals are available in S4–S6 Figs). The fitted games played function suggests that a draftee selected using pick one would be expected to play approximately 10% of the career games within their first season. This could be used as a proxy to discount future picks, where pick one from next year’s draft would be worth 10% less than its value today. Though the first hypothesis of a varying discount function was upheld, the rate seemed to increase with the draft. This could be due to the shorter careers of players selected at the tail end of the draft, increasing the proportion of game time they have in their first season compared to their overall careers. Individually the BMV function ranged between 2% and 4%, as they are awarded by on field umpires unlike games played or CMV and is subject to their own prejudices. Yet, the games played and CMV functions ran parallel to each other between 7% and 13%. However, the manner in which the CMV function lies below games played suggests that even if a recruit is on field, their effective contribution to a team might not necessarily correlate to their time [32], warranting a different approach to quantify this delay and create a more representative discount function.

### Difference between seasonal and career average performance per game

Whilst the previous model provides an understanding of how a discount function should look and the preference to use CMV derived metrics as opposed to games played and BMV, the results obtained were impacted by two key issues. First, the variation in career lengths across picks adversely affects latter selections as it increases their proportion of early career outcomes. Secondly, a player’s contribution in their first season might not necessarily have the same effect as their contribution later on in their careers. Hence, it is equally important to understand the returns expected within the context of a team. The performance derivatives used in Table 4, though illustrate the expected outcomes of a draftee in their first season when compared to their potential career, it does not articulate the need of the team.

Assuming that all teams aim to maximize wins (or aspire to build a team capable of delivering this outcome), a draftee’s potential to the roster is to consistently contribute to that effort. Hence, the derivative of the dependent variable used within the second model is the percentage difference between the draftee’s CMV in their first season and their career average throughout per game, thereby negating the career length effect and elevating the effectiveness of outcomes issue observed in the previous model. For example, if a draftee contributes six points to the margin of victory per game in their first season, and their career average contribution per game is 10, the new metric will assume a value of 40% ((10–6)/10). This essentially answers the question of discount in the team’s purview as it accounts for the time taken by the draftee to achieve the highest CMV per game. Table 5 furnishes the regression results of this new metric controlled for the effects as in the previous models.

**Table 5:** Percentage difference between specific seasons and career average CMV per game model.

Model Type	Linear Regression with Fixed Effects for Year Drafted							
Dependant Variable	Percentage Difference Between First Season and Career Average CMV per Game		Percentage Difference Between Second Season and Career Average CMV per Game		Percentage Difference Between Third Season and Career Average CMV per Game		Percentage Difference Between Fourth Season and Career Average CMV per Game	
Model Number	4		5		6		7	
Independent Variables	Coefficient	Robust Std. Err.	Coefficient	Robust Std. Err.	Coefficient	Robust Std. Err.	Coefficient	Robust Std. Err.
Drafting Age	-0.07***	0.02	-0.03**	0.01	-0.05**	0.02	-0.04**	0.01
Club academy								
≤ 2014	-0.18	0.12	-0.51***	0.07				
Father–son								
≤ 2006	0.01	0.14	-0.06	0.09	-0.12*	0.06	-0.07	0.07
2007 ≤ 2014	0.09	0.14	-0.04	0.12	-0.05	0.10	-0.01	0.06
Indigenous	-0.08	0.08	-0.11	0.08	-0.06	0.05	-0.06	0.07
Weight	0.01	0.01	0.01	0.01	0.01*	0.01	-0.01	0.01
Height	-0.01	0.01	0.01	0.01	0.01	0.01	0.01	0.01
Position Played ^a^								
Forward	-0.07*	0.04	0.03	0.05	-0.01	0.04	-0.01	0.05
Midfielder	-0.05	0.04	-0.01	0.05	0.01	0.03	-0.01	0.04
Ruckman	-0.02	0.06	-0.12	0.08	0.05	0.07	0.08	0.13
Drafting Team ^b^								
Brisbane	-0.36***	0.10	-0.29***	0.08	-0.21**	0.08	-0.15	0.10
Carlton	-0.26**	0.09	-0.18*	0.09	-0.34***	0.09	-0.16	0.11
Collingwood	-0.17	0.10	-0.25**	0.10	-0.19**	0.08	-0.07	0.08
Essendon	-0.25**	0.09	-0.31***	0.04	-0.15	0.12	-0.17**	0.06
Fremantle	-0.29**	0.11	-0.19**	0.06	-0.15	0.12	-0.03	0.07
Geelong	-0.01	0.07	0.02	0.09	-0.15	0.09	0.05	0.06
Gold Coast	-0.41***	0.08	-0.22**	0.08	-0.28**	0.12	-0.31***	0.04
Greater Western Sydney	-0.27**	0.11	-0.36***	0.07	-0.28***	0.08	-0.10	0.06
Hawthorn	-0.03	0.10	-0.05	0.08	0.03	0.15	-0.05	0.09
Melbourne	-0.15*	0.08	-0.27***	0.06	-0.23*	0.11	-0.14	0.08
North Melbourne	-0.13	0.11	-0.15*	0.07	-0.20**	0.08	-0.09	0.10
Port Adelaide	-0.07	0.08	-0.12	0.09	-0.08	0.08	-0.10*	0.05
Richmond	-0.18*	0.09	-0.23**	0.08	-0.08	0.08	-0.13	0.09
St. Kilda	-0.19	0.11	-0.24**	0.08	-0.12	0.13	-0.09	0.07
Sydney	-0.06	0.12	0.028	0.10	0.11	0.13	0.01	0.11
Westcoast	-0.21*	0.08	-0.17*	0.08	-0.24***	0.07	-0.13	0.09
Western Bulldogs	-0.16	0.12	-0.17*	0.08	-0.16	0.11	-0.14*	0.07
Amateur League ^c^								
SANFL	-0.07	0.05	0.12*	0.06	0.03	0.07	0.07	0.06
TAC Cup	-0.03	0.08	0.09*	0.05	-0.03	0.05	0.03	0.03
WAFL	0.03	0.07	0.16*	0.06	0.08	0.06	0.06	0.04
Constant	1.30	0.86	-0.70	0.64	-0.38	1.00	0.65	0.91
Draftees	689		573		451		370	
Listed Players Drafted between	2003–14		2003–13		2003–12		2003–11	
R-squared	0.12		0.14		0.18		0.08	
Prob(F-Statistic)	0.00		0.00		0.00		0.00	

^a^ the reference position is Defender

^b^ the reference team is Adelaide Crows

^c^ the reference league is Other.

*** p<0.01

** p<0.05

* p<0.1

The results in model 4 suggest the difference between a draftee’s first season average CMV per game and their overall career reduced by 7% as they get older. As mature players might have had more experience in other minor leagues, they might contribute more than their peers who are relatively young. Some drafting team indicators returned significant coefficients as well. For example, the difference between first season and career CMV per game for Fremantle draftees was 29% less when compared to the reference team of Adelaide, as they were exposed to more game time early on in their careers. The same effect was also observable amongst some other clubs, including Gold Coast which was one of only two examples in the previous model. When the model was repeated to predict the percentage difference between second (model 5), third (model 6), and fourth (model 7) season and career average CMV per game, Fremantle’s effect withered down as expected. Ideally a team would only retain players which it deems to effectively contribute to its ability to maximise wins. The significant coefficient for C/A observed in 5 could be attributed to the same effect observed in Table 4, as the career lengths of such draftees remains relatively shorter.

**Fig 4:**
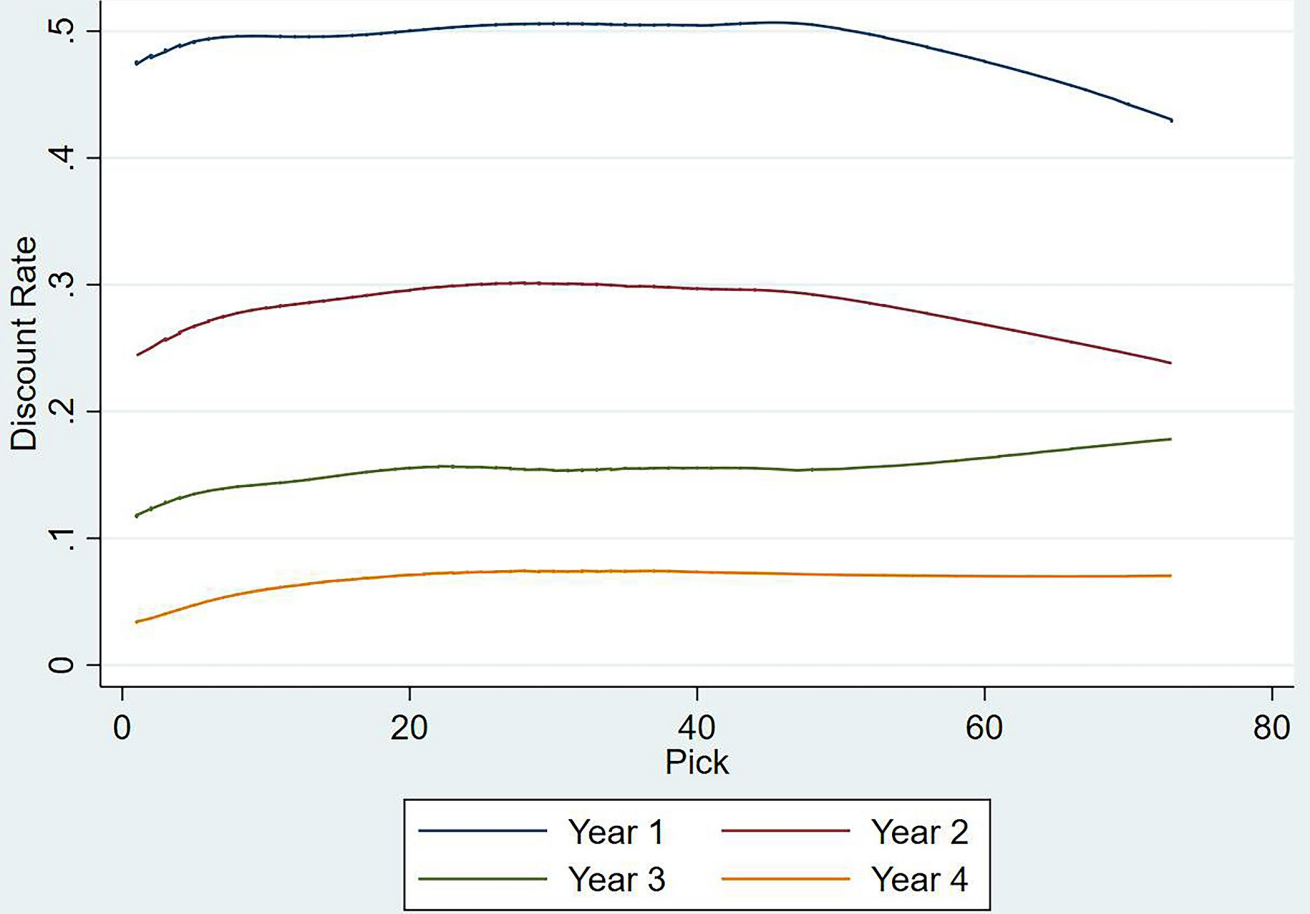
Expected percentage difference between specific seasons and career average CMV per game per pick.

In order to create the new discount function, the expected values from models 4, 5, 6 & 7 were used to fit a LOWESS curve in Fig 4 (the numerical values are available in S2 Table; the fitted plots including confidence intervals are available in S7–S10 Figs), with the discount rates hovering between 42% and 51%. For example, assuming pick one is equal to 100 points today, the same selection from next year’s draft would be worth 53 today (100*(1–0.47)). Unlike the previous models, the discount function derived here follows a somewhat concave curve, as opposed to the monotonically increasing versions. There is a slight increase observed amongst the initial picks, which could be the result of sunk investment plays [33] whereby decision makers tend to provide more on field time to recruits drafted early on, even when their on-field performance did not warrant that decision (similar to an investor riding losing investments and dumping winners [34]). More game time translates to quicker personal development, which in turn reduced their time to develop over their careers. Hence their average CMV per game in the first season might appear to be lesser than their inflated career potential. This further coincides with the suggestion that decision makers overvalue the initial picks in the draft [11] and retain these players beyond their marginal productivity levels [20, 35]. However, the discount function seems to increase in middle of the draft (between picks 20 and 40), contradicting our previous hypothesis. Previous studies have shown that picks from the middle of the draft are exchanged more frequently than both tail ends. This leads to the same escalation effects made evident with earlier picks, without the developmental uptick [20, 27] and larger dispersions in performance which in turn increases the discount rates. In the tail end of the draft, the discount rate declines again. This could either suggest that some talent is overlooked in the drafting process till later on (as suggested by the trend line in Fig 2, with sporadic observations of draftees denied any game time if they were picked early), or the shorter career lengths of later draftees puts their first season CMV per game to be more in par with their overall careers. The discount rate functions for years 2 and 4 followed a similar trend, whilst year three increased with the progression of the draft. After the end of the initial contract (at the end of year two), teams are most likely to retain players who align with their overall objectives in the long run. Unless tail end draftees show any potential, they might get delisted in this time and their outcomes within the team might be adversely skewed increasing their discount rate. When comparing the discount rates year-on-year, it is evident that the point-to-point difference between them reduced with each year as their experience grows.

### Pick value & application

**Fig 5:**
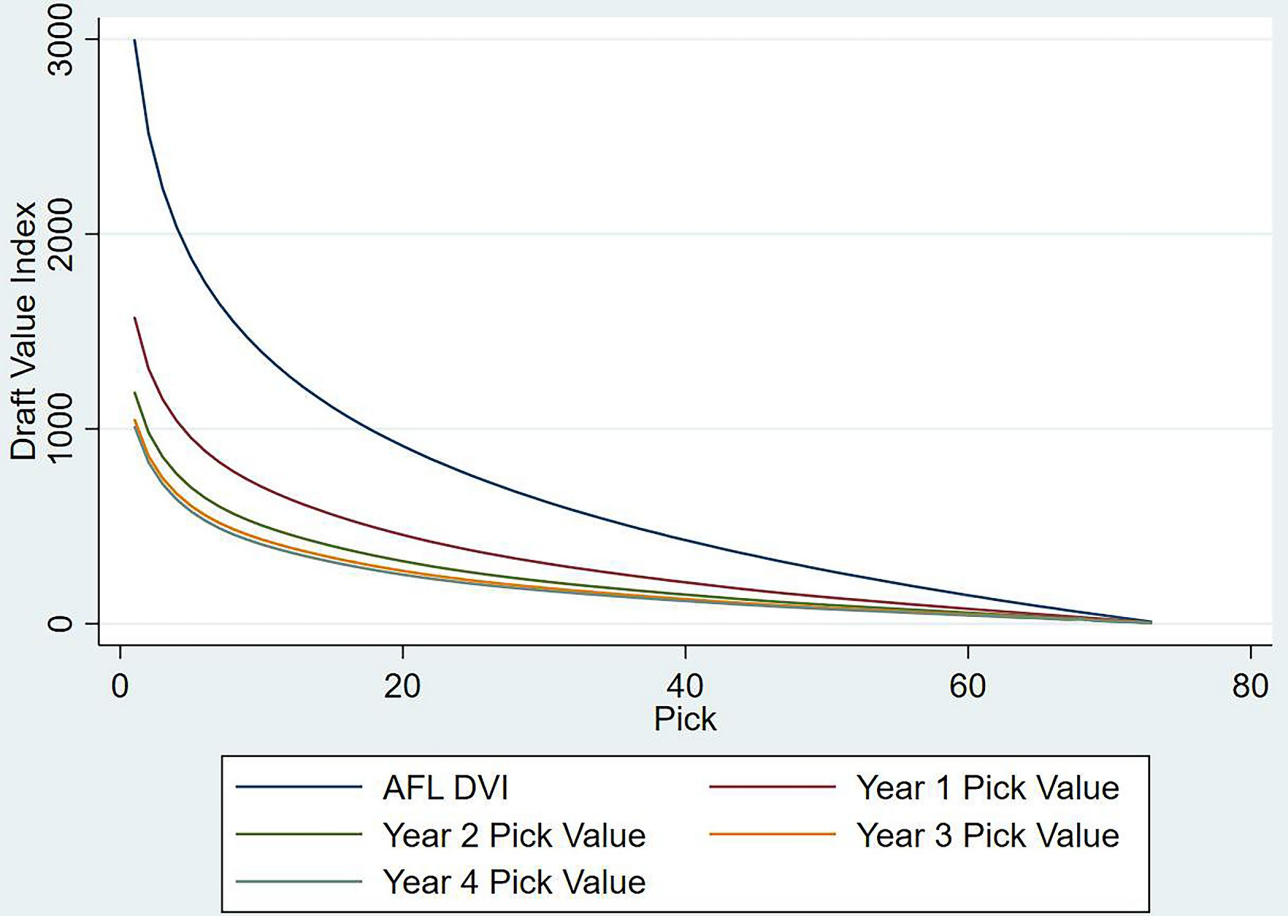
Current AFL DVI and discounted future pick value.

In order to overlay the discount functions estimated in the previous section, we chose the AFL’s Draft Value Index (DVI, [18]) as the basis for the value of a pick in the current year. This index and the respective values each year in the future was obtained by applying the discount rates presented in S2 Table and plotted in Fig 5 (the numerical values are available in S3 Table). Given the curvature of both the pick value and discount rate functions, the value of future picks loosely translated to varying picks in the current year. For example, the first pick next year was approximately equal to pick 8 in the current year, while pick one from year three matched pick 14 from the current year. Interestingly, the variance in pick value observed between the first and last selection reduced each year as only the best players would remain listed after their initial contract period.

In order to gauge the effectiveness of the proposed method, a pick swap between Hawthorn and St Kilda in 2016 has been presented in Table 6. In return for picks 10 and 68 of the 2016 draft, Hawthorn released picks 23 and 26 together with their first-round selection from 2017 to St Kilda. Without the proposed discount model, assuming that the teams use the same pick value method and expect parity in exchange (as done in previous studies [9, 10], where both teams exchange value that is equal, making no profit off one another and the total difference is attributed to the single future year pick in the trade), Hawthorn’s 2017 first round pick would be valued at 137 (1,395 + 59–815–502). Since, Hawthorn would not know their 2017 draft position in 2016 (eventually this was pick seven), this first round pick could lie anywhere between one and 18. If this is assumed to be pick one, for the trade to be equal, Hawthorn would have discounted this pick by 95.43% (1-137/3,000). Even at the last spot in round one (pick 18), the selection would need to be discounted by 86.09% (1-137/985) for the trade to be equal. However, through the discount rates introduced in this study, we could clearly understand that St Kilda made a profit (previously alluded to as implied discount in full) by deferring their right to choose in the current year, as the lowest value for next year’s round one pick (pick 18) is 494, which is higher than the difference of 137 mentioned earlier. This profit could be attributed to both, Hawthorn’s need for trading up and getting pick 10, as they may have endeavoured to use said selection to select an amateur player who was possibly in the prospective lists of multiple teams and their inability to predict their future standing (and by extension their position in next year’s draft).

**Table 6:** 2016 Trade between Hawthorn and St Kilda.

**Hawthorn received picks**				
10	1,395			
68	59			
	**1,454**			
**St Kilda received picks**				
23	815			
36	502			
	**1,317**			
***Difference***	**137**	2017 Hawthorn Round 1 (St Kilda Received)		
*Pick*	*Current DVI*	*Discount Rate* *(1-137/Current DVI)*	*Proposed Year One DVI*	*Proposed Discount Rates*
1	3,000	95.43%	1,575	47.49%
2	2,517	***94***.56%	1,309	47.99%
3	2,234	93.87%	1,152	48.45%
4	2,034	93.26%	1,041	48.83%
5	1,878	92.71%	955	49.15%
6	1,751	92.18%	886	49.38%
7	1,644	91.67%	830	49.52%
8	1,551	91.17%	782	49.60%
9	1,469	90.67%	740	49.62%
10	1,395	90.18%	703	49.61%
11	1,329	89.69%	670	49.59%
12	1,268	89.20%	639	49.57%
13	1,212	88.70%	611	49.56%
14	1,161	88.20%	585	49.57%
15	1,112	87.68%	560	49.61%
16	1,067	87.16%	537	49.66%
17	1,025	86.63%	515	49.73%
18	985	86.09%	494	49.82%

## Discussion

Trading picks is a common transaction observed in sporting leagues that employ player drafts [1]. However, many current and revised draft formats employed by professional leagues allow for the trading of picks in the current year for those in the future [4, 36]. This raises a question of an appropriate rate of discount which can be used to account for the delay in relinquishing current picks for those in the future. Using the difference between a draftee’s contribution to a team in their first season (second, third and fourth season for the subsequent discount rates) when compared to their overall career, this study created a discount rate function that can be used by decision makers within clubs to potentially evaluate trades involving draft picks from the future.

Referring back to the example put forward in the introduction, if team A wished to obtain team B’s fifth pick in the current year (valued at 1,878), they would need to release their pick nine in the current year (valued at 1,469) together with their first-round pick from the next year. At best, if team A finishes last in the ladder this year and obtain pick one next year, team B will make a significant profit of 1,166 DVI points in the trade (1,575 (pick one next year) + 1,469–1,878) and at worst, breakeven with a slight margin of 85 (494 (pick 18 next year) + 1,469–1,878). Whilst the proposed metric might not necessarily allow teams to understand the exact value exchanged in each trade at the time it was executed due to the inability to predict next year’s drafting order, it would allow them to hedge themselves according to their risk tolerances. Going back to the previous example, if team B was content with breaking even (not making a profit) on the trade, team A could forgo its second-round pick from next year instead of its first-round pick. However, this pick has to be between 19 and 23 (i.e., team A has to be placed between 14 and 18 in the ladder next year) for team B to breakeven as any lower could put it in a net negative. Though this could be achievable based on a number of factors, team B might not be willing to risk it and would ideally prefer a first-round pick to guarantee a net positive result.

**Fig 6:**
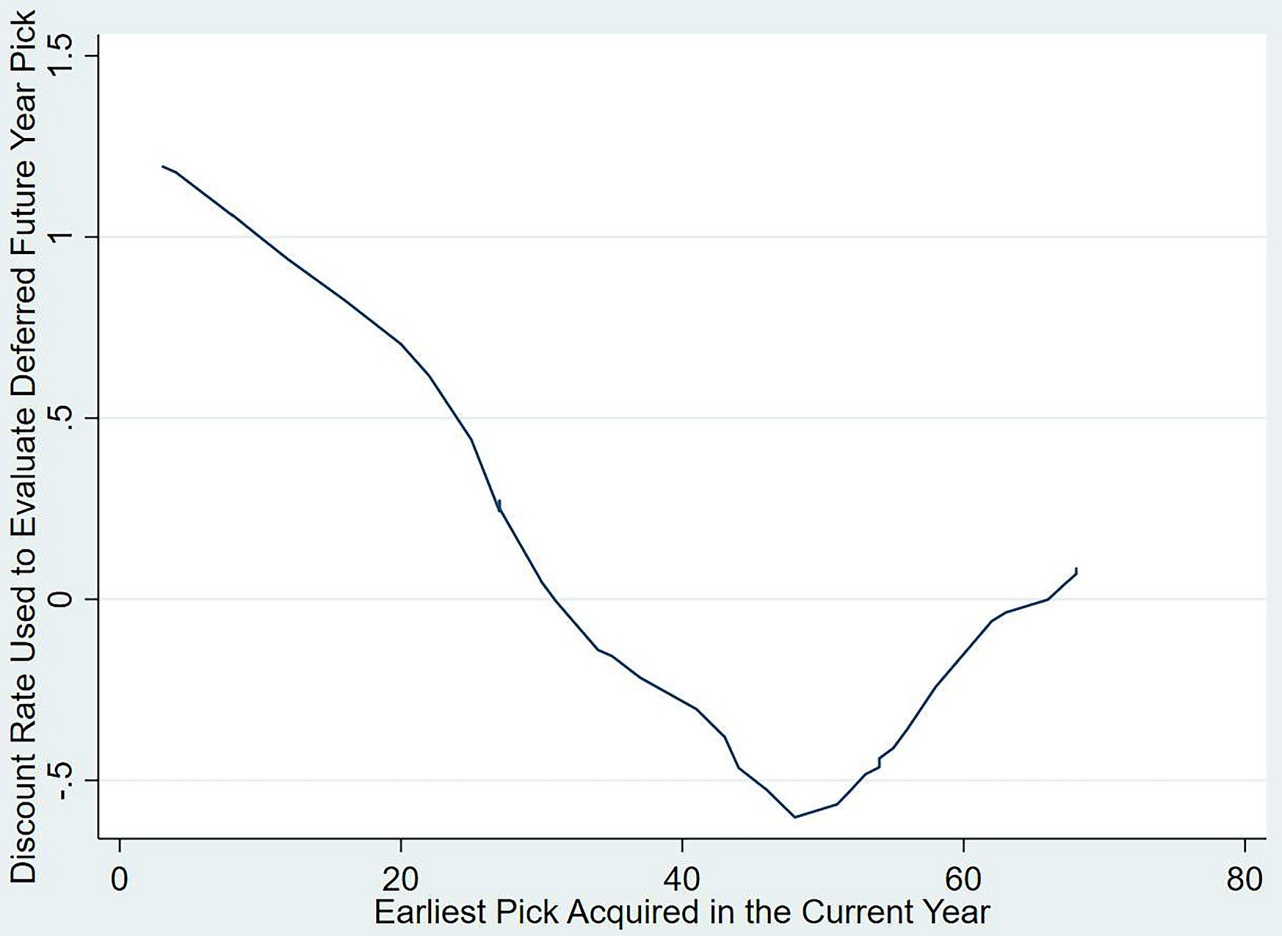
Discount rate used to evaluate deferred future picks plotted against the earliest pick acquired today in single future pick trades between 2015 and 2022.

Another aspect motivating teams when evaluating trades is the urgency of the earliest pick to the team trading up (giving up later picks for the early selection). As mentioned in the previous section with the Hawthorn-St Kilda example, it is clearly visible that St Kilda made a profit in this trade by trading down (obtaining later selections in the 2016 draft and a deferred choice in the 2017 lineup). However, Hawthorn’s motivations to give up this profit could be due to their want to secure a key player using pick 10. Using all the trade data available between 2015 and 2016 where a selection of current year picks was exchanged together with one future year selection, Fig 6 plotted a LOWESS function between the implied discount rate (where the trade is expected to be equal) attributed to the future pick and the earliest selection in the trade. For example, to represent Port Adelaide receiving pick 12 in the 2021 draft, by releasing pick 14 and their second-round pick from 2022 to West Coast, the figure would create plot against pick 12 in the horizontal axis (as it is the earliest selection). For the vertical axis, the difference of 107 (1,268–1,161) between picks 12 and 14 would then be compared against the value of pick 28 (677 in the DVI), which is the expected second-round pick of Port Adelaide in 2022 (as per www.draftguru.com). This would amount to 84% (1-(107/677)). With previous studies suggesting trading down (giving up early picks for a few later on) creates opportunities for arbitrage as teams over value early selections [9, 11], the behaviour observed in the AFL future pick trading market also validated this argument. Should teams opt to heavily discount future picks to trade up to the current year, decision makers could create an opportunity for arbitrage by deferring their picks (which is essentially another form of trading down). The trend observed in Fig 6 further supplemented the proposition made by the previous authors. Essentially a team with a prospect of obtaining the first pick in a trade could discount a future pick, that it could relinquish as part of the negotiation, by up to 120%. This clearly portrays the team’s need for instant gratification over deferred options [37–39], ‘because the present is valued discretely more than the future’ [40]. However, this rate declines and goes into the negative as the earliest pick in a trade reaches 30, whereby managers hedge their bets in seeking out better talent options in the future.

## Limitations

Whilst this study provides decision makers with a discount rate which could be potentially used to evaluate trades involving future picks, the functions themselves were based on derivatives of performance (percentage difference between specific seasons and career average CMV per game). These performance derivatives could be influenced by a variety of factors, including but not limited to, a player’s physical structure, position played, amateur league experience, race, drafting team, injury periods, movements between rookie and senior lists, and player trades [11, 17]. Though the predicted estimates used to fit the LOWESS function controls for a few of these factors, it is reasonable to assume that these might not be fully eliminated. Coupled with the escalation effects discussed early on, the deviation of the discount rate per pick from the mean could be influenced by factors not necessarily related to player performance. Whilst the proposed discount rates are derived on expected performance, they have been overlaid on the AFL DVI to project future values of draft picks. If this is replaced with a different value function such as those available in the current literature, the basis of analysis would shift leading to different outcomes. Furthermore, the inability to predict future draft order makes teams that forgo current picks in return for future ones err on the side of caution and hedge their bets. This could explain the reliance of decision makers on market rates to evaluate trades (for both current and future exchanges) even though the current literature hosts a variety of works valuing picks based on player performance [2, 3, 15, 17].

## Conclusion

Evaluating multi-year investment propositions is an arduous task, especially when the returns are of a non-monetary nature. Yet such decisions are entertained by list managers in many professional sporting leagues with restrictive labour markets. Though player drafts are intended to disperse amateur talent equitably, it creates a broad range of alternative outcomes that ought to be evaluated on its own merits. One such alternative is the right to defer or advance your draft pick through trades.

Ideally, for trades to always have parity in exchange, accurate denominations of value are required. Whilst the prevailing literature provides alternative methods to value picks and players to evaluate such trades, the question of future selections remained unanswered. As the expected outcomes of draftees vary based on the pick used to recruit the player, this study proposed a discount rate function within the context of the AFL. By overlaying this into the AFL’s DVI, it provides AFL clubs and list managers with a potential approach in evaluating trades involving future picks. The findings held the hypotheses of a variable discount rate that drops with the progression of the draft. Though the AFL only allows trading of picks up to one year in the future (to be increased to two in 2025 [19]), this model has shown its versatility to be retrofitted to the needs of other North American drafts which allows trades involving picks up to four years into the future.

## Supporting information

S1 Fig Frequency of draftees per career games played interval. (TIF)

S2 Fig Frequency of draftees per career BMV interval. (TIF)

S3 Fig Frequency of draftees per career CMV interval. (TIF)

S4 Fig Fitted values from model 1 together with LOWESS. (TIF)

S5 Fig Fitted values from model 2 together with LOWESS. (TIF)

S6 Fig Fitted values from model 3 together with LOWESS. (TIF)

S7 Fig Fitted values from model 4 together with LOWESS. (TIF)

S8 Fig Fitted values from model 5 together with LOWESS. (TIF)

S9 Fig Fitted values from model 6 together with LOWESS. (TIF)

S10 Fig Fitted values from model 7 together with LOWESS. (TIF)

S1 Table CMV predictor. (DOCX)

S2 Table Predicted discount rates per pick based on models 4–7. (DOCX)

S3 Table AFL DVI with discounted rates for future picks. (DOCX)

S1 Data Data used in the study. (XLSX)
