# Supplementary figures and images for: Deferring draft picks: Empirical analysis of the AFL draft
Source: PLoS One. 2024 Sep 27;19(9):e0311240. doi: 10.1371/journal.pone.0311240 (PMC11433144; doi:10.1371/journal.pone.0311240)

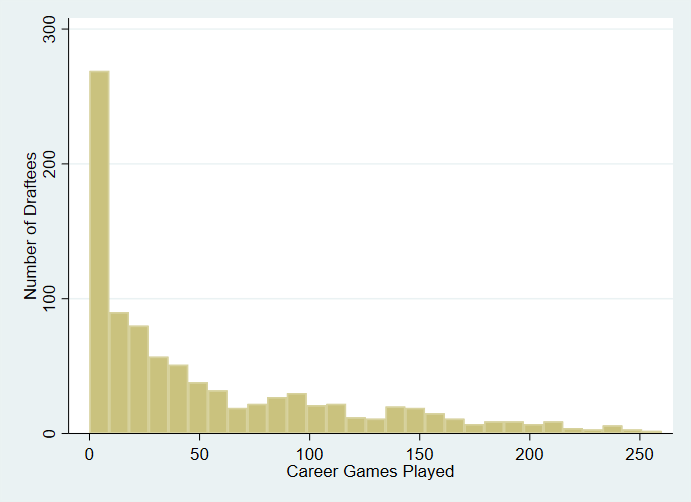

Supplement: S1 Fig — (TIF) [file pone.0311240.s001.tif]

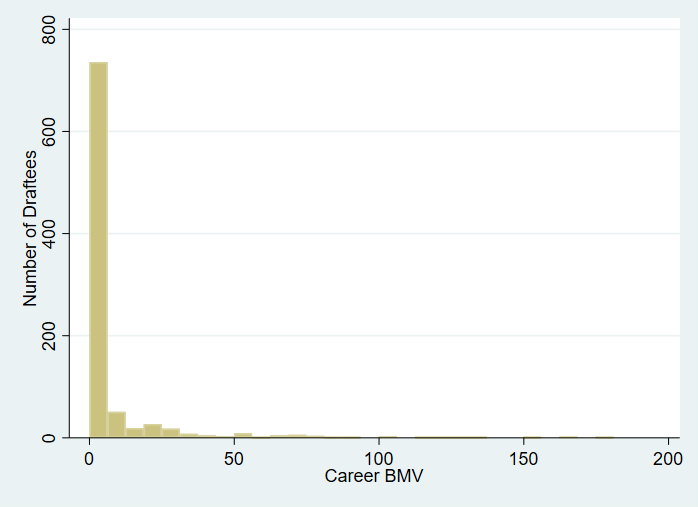

Supplement: S2 Fig — (TIF) [file pone.0311240.s002.tif]

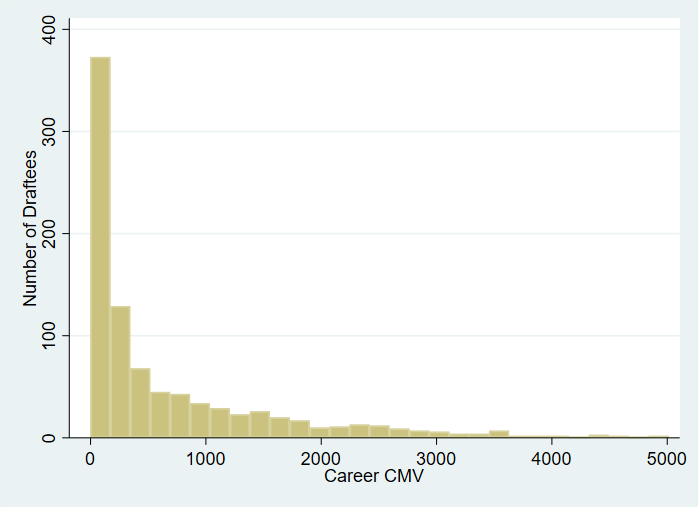

Supplement: S3 Fig — (TIF) [file pone.0311240.s003.tif]

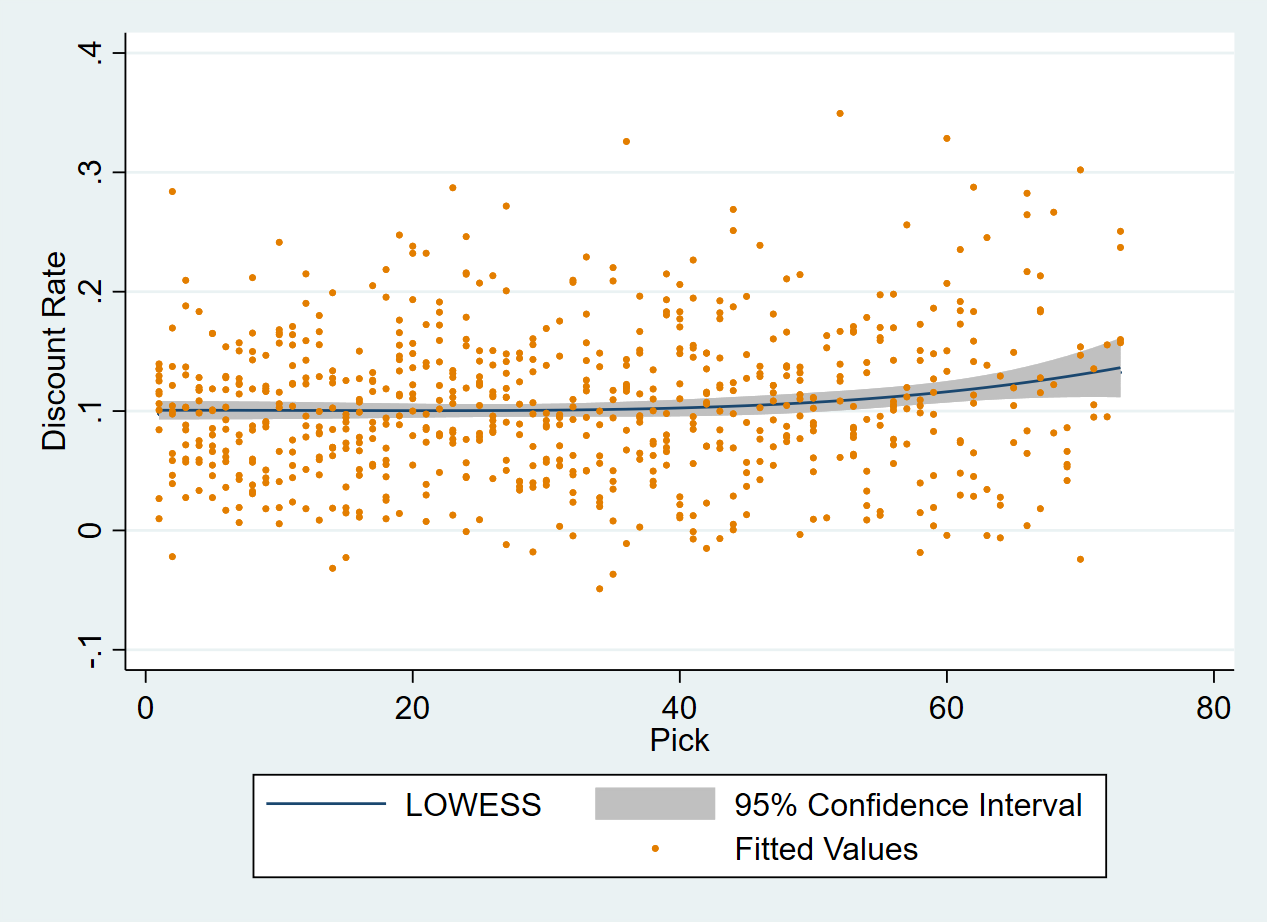

Supplement: S4 Fig — (TIF) [file pone.0311240.s004.tif]

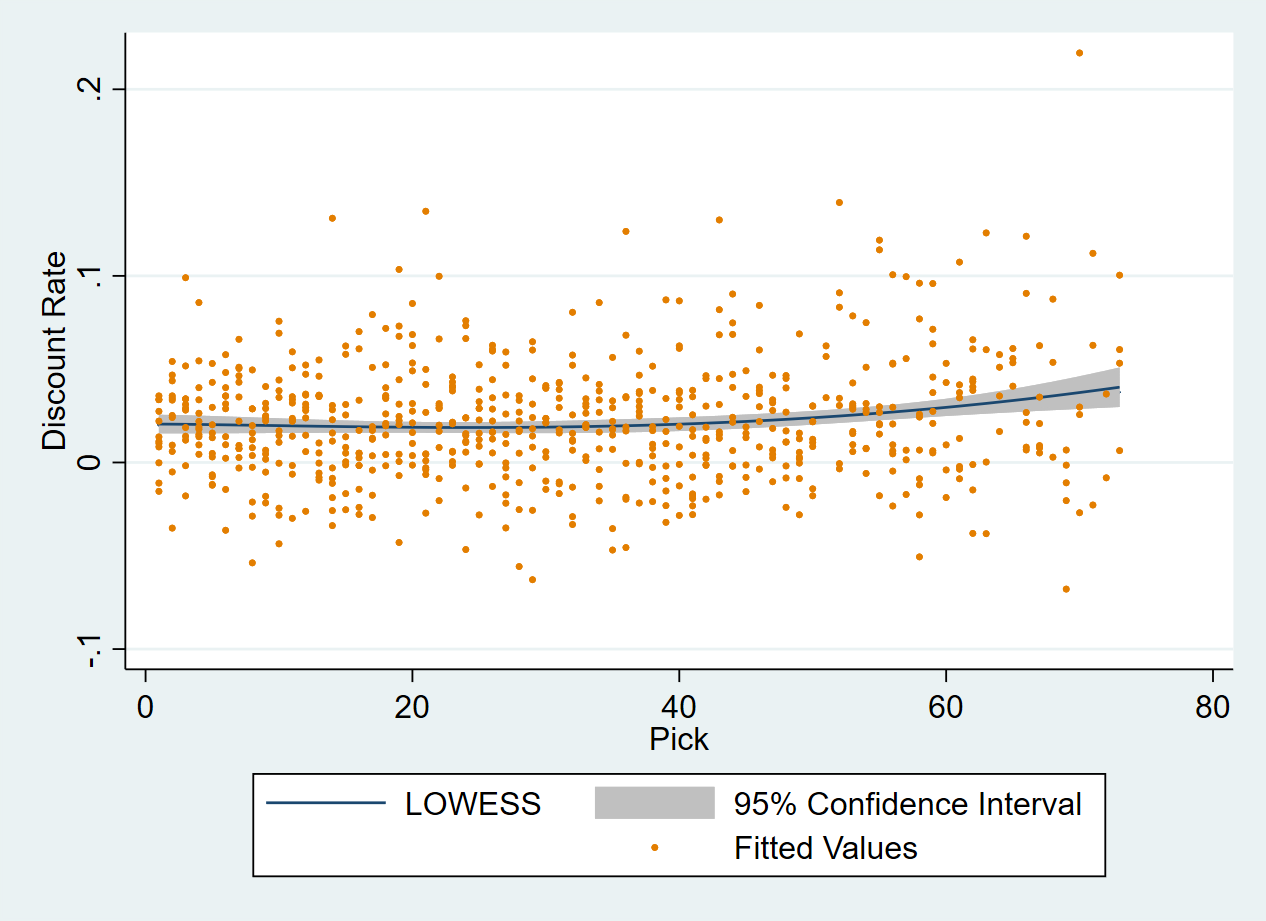

Supplement: S5 Fig — (TIF) [file pone.0311240.s005.tif]

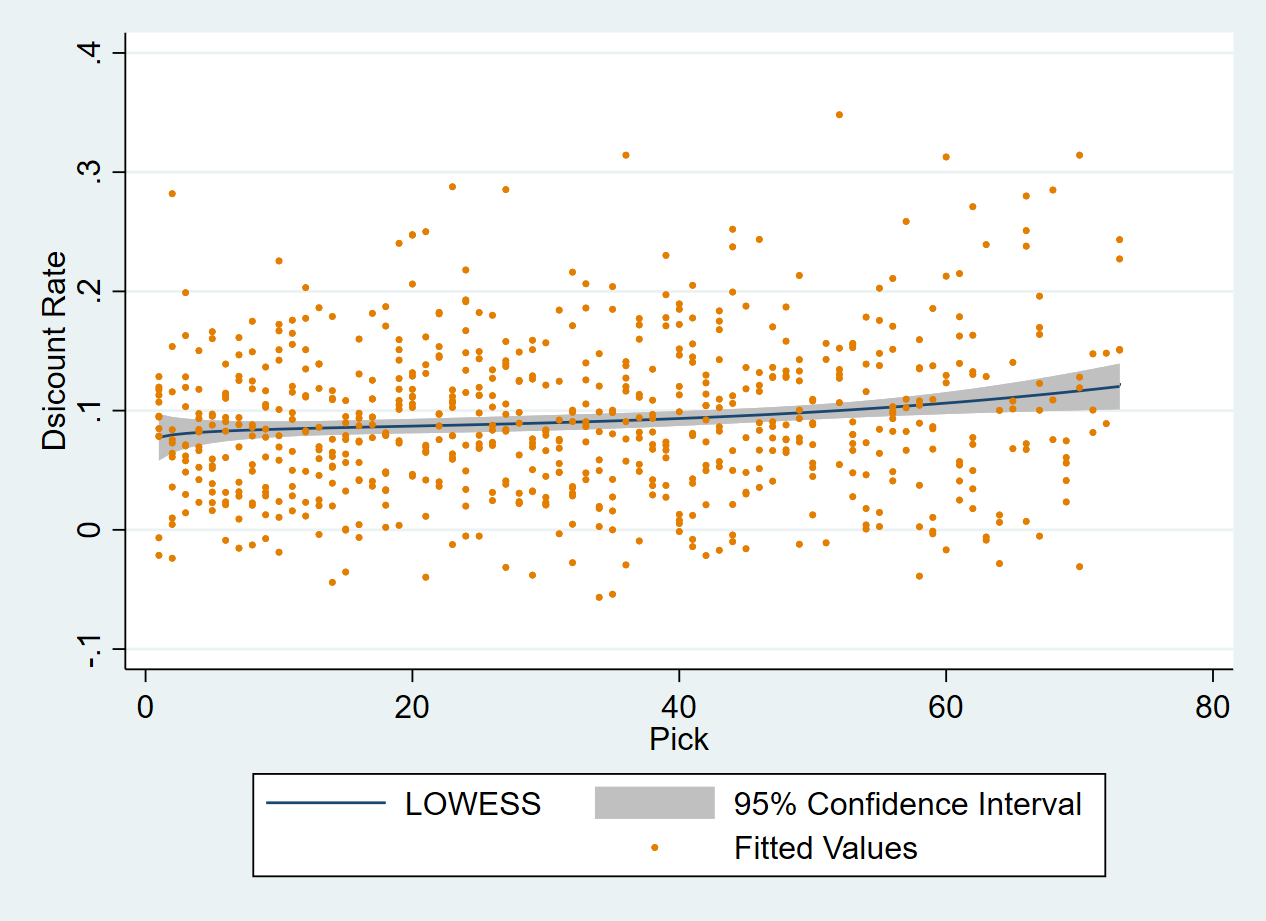

Supplement: S6 Fig — (TIF) [file pone.0311240.s006.tif]

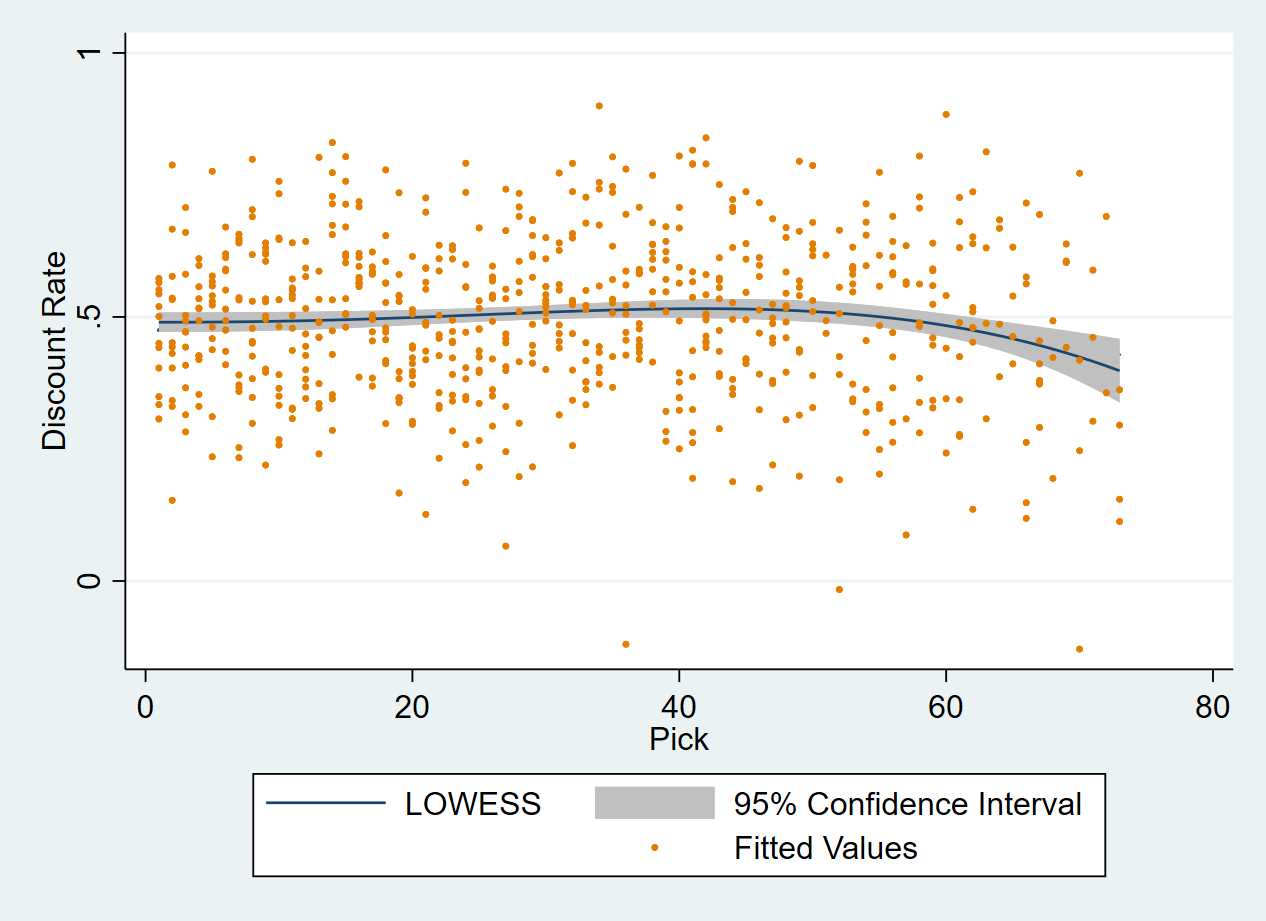

Supplement: S7 Fig — (TIF) [file pone.0311240.s007.tif]

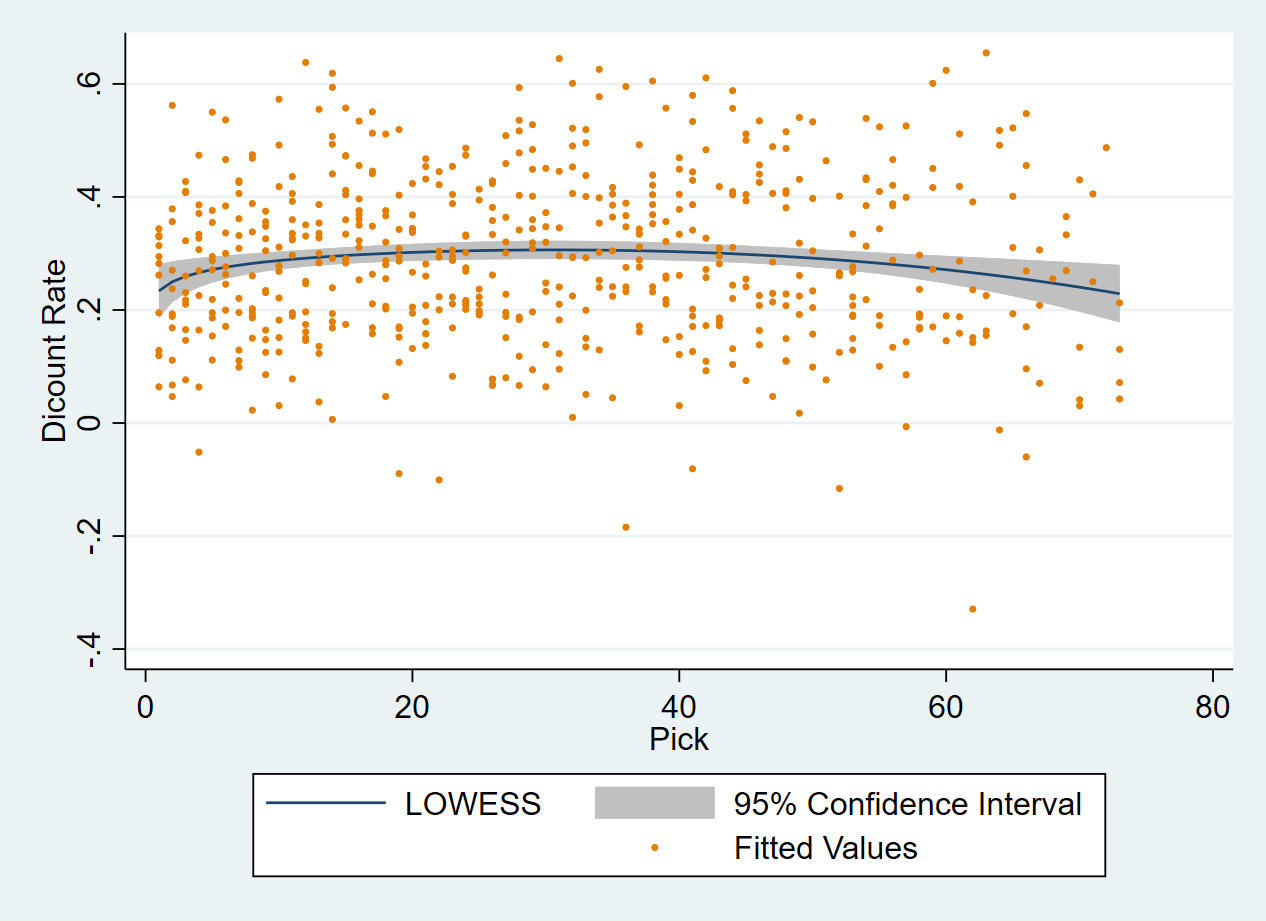

Supplement: S8 Fig — (TIF) [file pone.0311240.s008.tif]

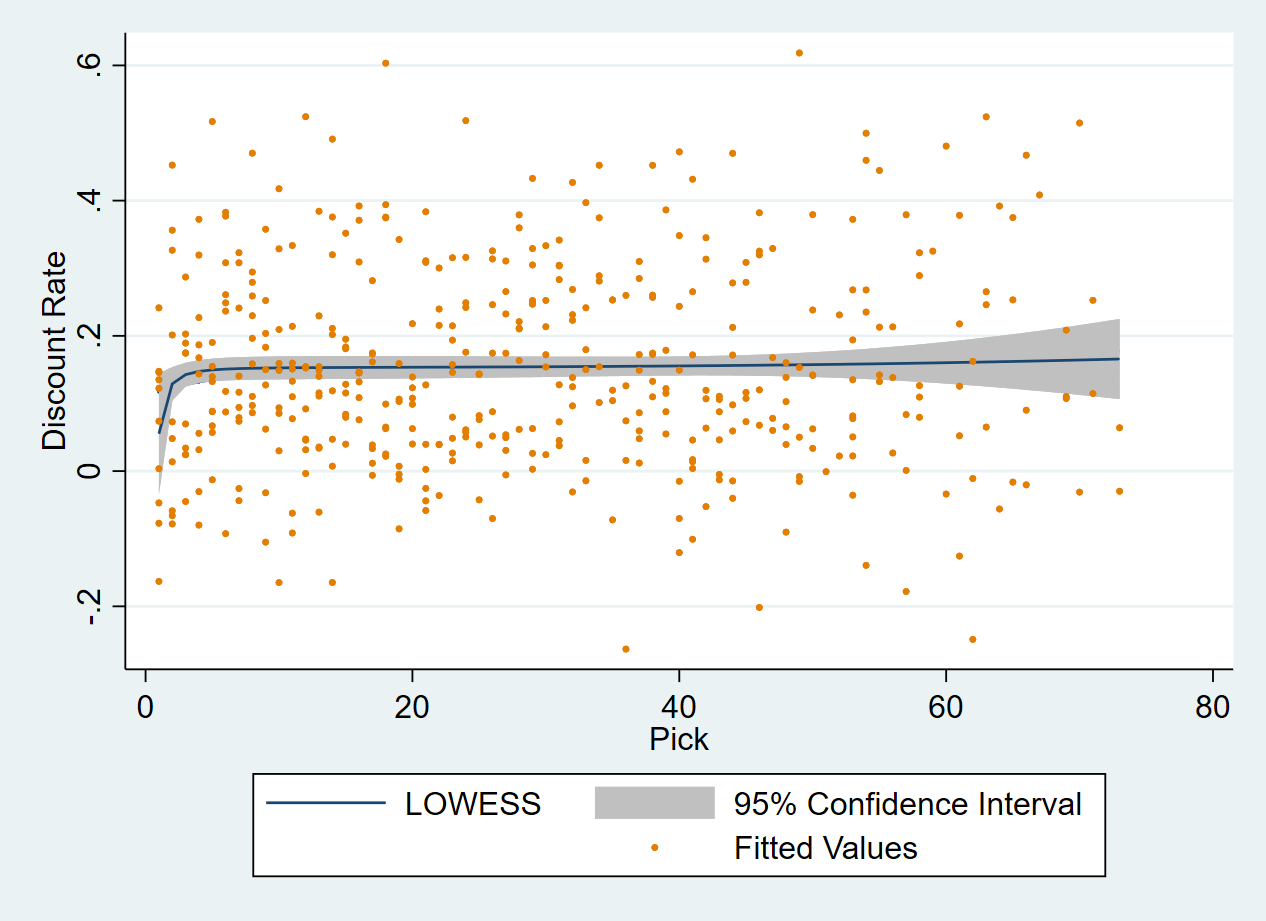

Supplement: S9 Fig — (TIF) [file pone.0311240.s009.tif]

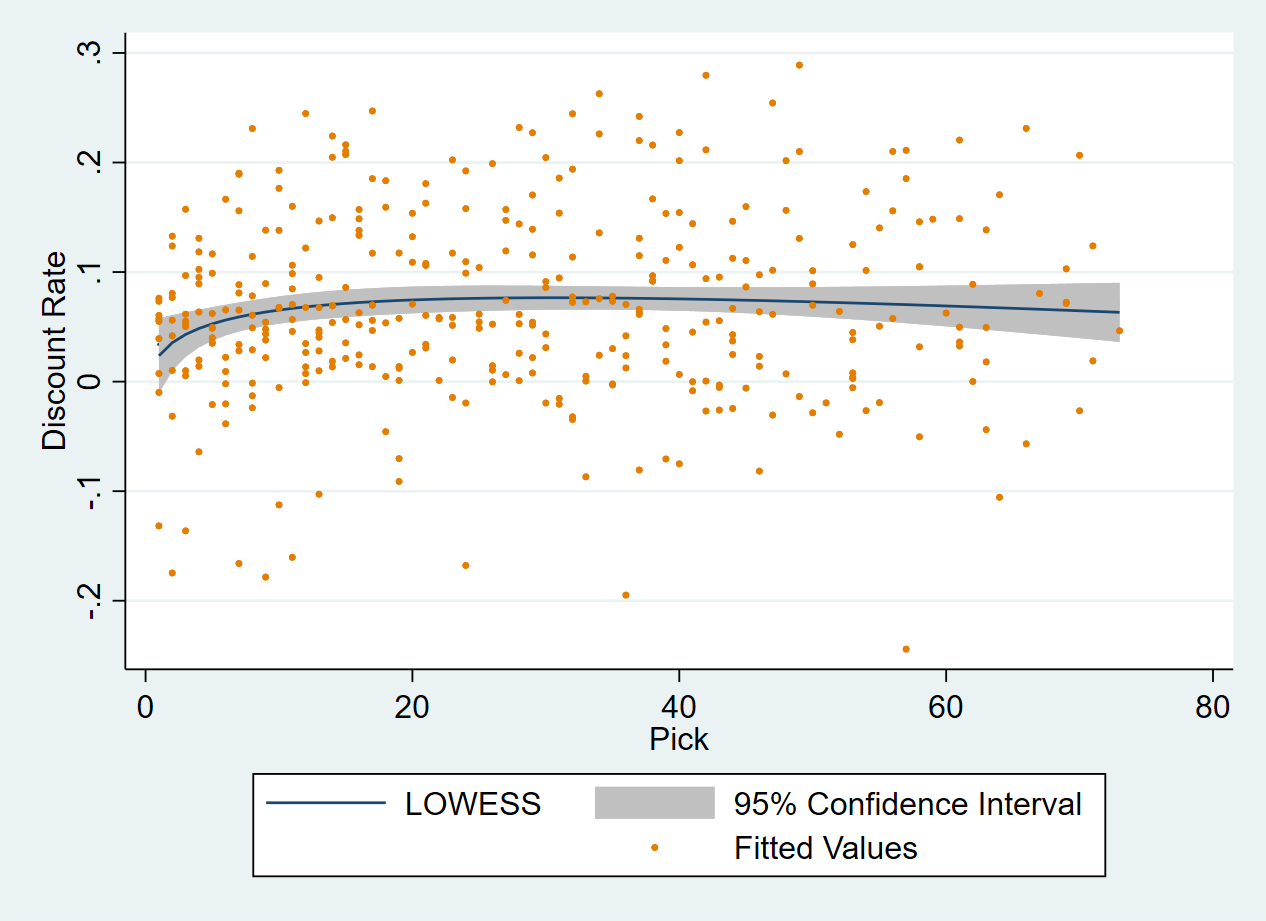

Supplement: S10 Fig — (TIF) [file pone.0311240.s010.tif]
