# Supplementary material for: Deferring draft picks: Empirical analysis of the AFL draft
Source: PLoS One. 2024 Sep 27;19(9):e0311240. doi: 10.1371/journal.pone.0311240 (PMC11433144; doi:10.1371/journal.pone.0311240)
Supplement: S1 Table — (DOCX) [file pone.0311240.s011.docx]

| Model Variables | Ordinary Least Squares | | | | |
| --- | --- | --- | --- | --- | --- |
|  | Winning Team Margin of Victory | | | | |
|  | [8] | |  | [9] | |
|  | Coefficient | Std. Err. |  | Coefficient | Std. Err. |
| Kicks | 0.11*** | 0.02 |  | 0.11*** | 0.02 |
| Marks | -0.08*** | 0.03 |  | -0.08*** | 0.03 |
| Contested Marks | 0.32*** | 0.06 |  | 0.31*** | 0.05 |
| Effective Possessions | -0.02* | 0.01 |  | -0.01** | 0.01 |
| Contested Possessions | 0.21*** | 0.03 |  | 0.21*** | 0.03 |
| Uncontested Possessions | 0.00 | 0.01 |  |  |  |
| Hitouts | -0.02 | 0.01 |  |  |  |
| Tackles | 0.09*** | 0.02 |  | 0.09*** | 0.02 |
| Rebounds | 2.48*** | 0.06 |  | 2.48*** | 0.06 |
| Inside 50s’ | 2.63*** | 0.06 |  | 2.63*** | 0.06 |
| Clearances | -0.06* | 0.03 |  | -0.06* | 0.03 |
| Clangers | -0.44*** | 0.03 |  | -0.44*** | 0.03 |
| Bounces | 0.054** | 0.03 |  | 0.06** | 0.03 |
| Marks Inside 50 | 0.18*** | 0.05 |  | 0.18*** | 0.05 |
| Assists | 2.81*** | 0.07 |  | 2.81*** | 0.07 |
| Frees For | 0.91 | 0.60 |  |  |  |
| Frees Against | 1.26** | 0.60 |  | 0.35*** | 0.05 |
| One Percenters | 0.13*** | 0.02 |  | 0.13*** | 0.02 |
| Constant | -0.27 | 0.24 |  | -0.27 | 0.24 |
| Observations | 2,918 |  |  | 2,918 |  |
| Adjusted R-squared | 0.92 |  |  | 0.93 |  |
| F-Statistic | 1,981.31 |  |  | 2,376.62 |  |
| Prob(F-Statistic) | 0.00 |  |  | 0.00 |  |
| *** p<0.01, ** p<0.05, * p<0.1 | | | | | |
