# Supplementary material for: Deferring draft picks: Empirical analysis of the AFL draft
Source: PLoS One. 2024 Sep 27;19(9):e0311240. doi: 10.1371/journal.pone.0311240 (PMC11433144; doi:10.1371/journal.pone.0311240)
Supplement: S2 Table — (DOCX) [file pone.0311240.s012.docx]

| Pick | Year 1 | Year 2 | Year 3 | Year 4 |
| --- | --- | --- | --- | --- |
| 1 | 47.49% | 24.43% | 11.78% | 3.41% |
| 2 | 47.99% | 25.02% | 12.32% | 3.69% |
| 3 | 48.45% | 25.65% | 12.79% | 4.04% |
| 4 | 48.83% | 26.20% | 13.17% | 4.39% |
| 5 | 49.15% | 26.70% | 13.49% | 4.74% |
| 6 | 49.38% | 27.12% | 13.73% | 5.05% |
| 7 | 49.52% | 27.48% | 13.92% | 5.33% |
| 8 | 49.60% | 27.76% | 14.06% | 5.57% |
| 9 | 49.62% | 27.99% | 14.17% | 5.78% |
| 10 | 49.61% | 28.17% | 14.27% | 5.96% |
| 11 | 49.59% | 28.32% | 14.38% | 6.13% |
| 12 | 49.57% | 28.45% | 14.49% | 6.28% |
| 13 | 49.56% | 28.59% | 14.63% | 6.42% |
| 14 | 49.57% | 28.72% | 14.78% | 6.54% |
| 15 | 49.61% | 28.86% | 14.93% | 6.65% |
| 16 | 49.66% | 29.01% | 15.09% | 6.75% |
| 17 | 49.73% | 29.16% | 15.23% | 6.84% |
| 18 | 49.82% | 29.31% | 15.35% | 6.94% |
| 19 | 49.91% | 29.45% | 15.46% | 7.02% |
| 20 | 50.03% | 29.58% | 15.55% | 7.12% |
| 21 | 50.13% | 29.70% | 15.62% | 7.17% |
| 22 | 50.23% | 29.80% | 15.64% | 7.24% |
| 23 | 50.31% | 29.91% | 15.65% | 7.26% |
| 24 | 50.39% | 29.98% | 15.62% | 7.32% |
| 25 | 50.46% | 30.03% | 15.61% | 7.34% |
| 26 | 50.51% | 30.10% | 15.57% | 7.37% |
| 27 | 50.55% | 30.12% | 15.51% | 7.39% |
| 28 | 50.57% | 30.13% | 15.44% | 7.42% |
| 29 | 50.58% | 30.11% | 15.43% | 7.38% |
| 30 | 50.59% | 30.09% | 15.37% | 7.39% |
| 31 | 50.59% | 30.07% | 15.35% | 7.38% |
| 32 | 50.58% | 30.04% | 15.37% | 7.40% |
| 33 | 50.56% | 30.03% | 15.40% | 7.40% |
| 34 | 50.53% | 29.96% | 15.43% | 7.41% |
| 35 | 50.51% | 29.89% | 15.51% | 7.40% |
| 36 | 50.50% | 29.88% | 15.51% | 7.41% |
| 37 | 50.49% | 29.85% | 15.54% | 7.41% |
| 38 | 50.50% | 29.80% | 15.54% | 7.40% |
| 39 | 50.48% | 29.73% | 15.54% | 7.37% |
| 40 | 50.46% | 29.69% | 15.55% | 7.34% |
| 41 | 50.48% | 29.65% | 15.55% | 7.31% |
| 42 | 50.53% | 29.63% | 15.56% | 7.29% |
| 43 | 50.59% | 29.62% | 15.54% | 7.27% |
| 44 | 50.65% | 29.59% | 15.53% | 7.25% |
| 45 | 50.68% | 29.54% | 15.49% | 7.23% |
| 46 | 50.67% | 29.47% | 15.43% | 7.20% |
| 47 | 50.62% | 29.37% | 15.35% | 7.17% |
| 48 | 50.50% | 29.23% | 15.41% | 7.15% |
| 49 | 50.35% | 29.08% | 15.44% | 7.14% |
| 50 | 50.18% | 28.92% | 15.47% | 7.11% |
| 51 | 49.97% | 28.74% | 15.55% | 7.10% |
| 52 | 49.75% | 28.55% | 15.62% | 7.09% |
| 53 | 49.51% | 28.36% | 15.67% | 7.08% |
| 54 | 49.26% | 28.15% | 15.74% | 7.07% |
| 55 | 49.01% | 27.95% | 15.83% | 7.06% |
| 56 | 48.74% | 27.73% | 15.91% | 7.05% |
| 57 | 48.47% | 27.51% | 16.02% | 7.04% |
| 58 | 48.19% | 27.29% | 16.12% | 7.03% |
| 59 | 47.91% | 27.07% | 16.24% | 7.02% |
| 60 | 47.61% | 26.85% | 16.34% | 7.02% |
| 61 | 47.31% | 26.63% | 16.45% | 7.01% |
| 62 | 47.01% | 26.40% | 16.57% | 7.01% |
| 63 | 46.70% | 26.18% | 16.67% | 7.00% |
| 64 | 46.38% | 25.95% | 16.82% | 7.00% |
| 65 | 46.07% | 25.72% | 16.92% | - |
| 66 | 45.73% | 25.50% | 17.05% | 7.00% |
| 67 | 45.38% | 25.26% | 17.17% | 7.00% |
| 68 | 45.00% | 25.03% | - | - |
| -69 | 44.65% | 24.80% | 17.39% | 7.01% |
| 70 | 44.23% | 24.55% | 17.50% | 7.02% |
| 71 | 43.82% | 24.32% | 17.62% | 7.03% |
| 72 | 43.43% | 24.08% | - | - |
| 73 | 42.95% | 23.80% | 17.82% | 7.04% |
