# Supplementary material for: Deferring draft picks: Empirical analysis of the AFL draft
Source: PLoS One. 2024 Sep 27;19(9):e0311240. doi: 10.1371/journal.pone.0311240 (PMC11433144; doi:10.1371/journal.pone.0311240)
Supplement: S3 Table — (DOCX) [file pone.0311240.s013.docx]

| Pick | Current AFL DVI | Year 1 | Year 2 | Year 3 | Year 4 |
| --- | --- | --- | --- | --- | --- |
| 1 | 3,000.00 | 1,575.40 | 1,190.57 | 1,050.36 | 1,014.56 |
| 2 | 2,517.00 | 1,309.05 | 981.48 | 860.60 | 828.87 |
| 3 | 2,234.00 | 1,151.72 | 856.36 | 746.84 | 716.70 |
| 4 | 2,034.00 | 1,040.76 | 768.07 | 666.89 | 637.58 |
| 5 | 1,878.00 | 954.88 | 699.95 | 605.50 | 576.77 |
| 6 | 1,751.00 | 886.36 | 645.96 | 557.24 | 529.08 |
| 7 | 1,644.00 | 829.85 | 601.83 | 518.06 | 490.47 |
| 8 | 1,551.00 | 781.77 | 564.71 | 485.30 | 458.28 |
| 9 | 1,469.00 | 740.13 | 532.99 | 457.47 | 431.03 |
| 10 | 1,395.00 | 702.98 | 504.96 | 432.88 | 407.06 |
| 11 | 1,329.00 | 670.01 | 480.27 | 411.21 | 386.00 |
| 12 | 1,268.00 | 639.50 | 457.53 | 391.22 | 366.66 |
| 13 | 1,212.00 | 611.33 | 436.56 | 372.69 | 348.75 |
| 14 | 1,161.00 | 585.47 | 417.30 | 355.61 | 332.35 |
| 15 | 1,112.00 | 560.38 | 398.64 | 339.10 | 316.54 |
| 16 | 1,067.00 | 537.14 | 381.32 | 323.80 | 301.93 |
| 17 | 1,025.00 | 515.26 | 365.01 | 309.43 | 288.26 |
| 18 | 985.00 | 494.31 | 349.44 | 295.79 | 275.26 |
| 19 | 948.00 | 474.81 | 334.98 | 283.18 | 263.30 |
| 20 | 912.00 | 455.75 | 320.95 | 271.06 | 251.77 |
| 21 | 878.00 | 437.86 | 307.82 | 259.74 | 241.11 |
| 22 | 845.00 | 420.59 | 295.25 | 249.08 | 231.05 |
| 23 | 815.00 | 404.96 | 283.84 | 239.40 | 222.01 |
| 24 | 785.00 | 389.44 | 272.70 | 230.11 | 213.27 |
| 25 | 756.00 | 374.55 | 262.07 | 221.16 | 204.93 |
| 26 | 729.00 | 360.82 | 252.22 | 212.96 | 197.27 |
| 27 | 703.00 | 347.66 | 242.95 | 205.27 | 190.10 |
| 28 | 677.00 | 334.65 | 233.83 | 197.73 | 183.05 |
| 29 | 653.00 | 322.68 | 225.52 | 190.72 | 176.64 |
| 30 | 629.00 | 310.82 | 217.31 | 183.91 | 170.32 |
| 31 | 606.00 | 299.45 | 209.41 | 177.26 | 164.18 |
| 32 | 584.00 | 288.60 | 201.92 | 170.88 | 158.24 |
| 33 | 563.00 | 278.36 | 194.78 | 164.78 | 152.59 |
| 34 | 542.00 | 268.11 | 187.79 | 158.81 | 147.05 |
| 35 | 522.00 | 258.33 | 181.12 | 153.03 | 141.70 |
| 36 | 502.00 | 248.51 | 174.27 | 147.23 | 136.32 |
| 37 | 483.00 | 239.16 | 167.76 | 141.70 | 131.20 |
| 38 | 465.00 | 230.19 | 161.61 | 136.50 | 126.40 |
| 39 | 446.00 | 220.87 | 155.20 | 131.08 | 121.42 |
| 40 | 429.00 | 212.55 | 149.45 | 126.21 | 116.95 |
| 41 | 412.00 | 204.04 | 143.55 | 121.23 | 112.37 |
| 42 | 395.00 | 195.41 | 137.51 | 116.12 | 107.66 |
| 43 | 378.00 | 186.76 | 131.45 | 111.01 | 102.94 |
| 44 | 362.00 | 178.65 | 125.78 | 106.25 | 98.54 |
| 45 | 347.00 | 171.14 | 120.58 | 101.91 | 94.54 |
| 46 | 331.00 | 163.29 | 115.17 | 97.40 | 90.39 |
| 47 | 316.00 | 156.05 | 110.22 | 93.30 | 86.61 |
| 48 | 302.00 | 149.48 | 105.79 | 89.48 | 83.08 |
| 49 | 287.00 | 142.48 | 101.05 | 85.45 | 79.35 |
| 50 | 273.00 | 136.00 | 96.67 | 81.71 | 75.90 |
| 51 | 259.00 | 129.58 | 92.34 | 77.99 | 72.45 |
| 52 | 246.00 | 123.60 | 88.31 | 74.52 | 69.24 |
| 53 | 233.00 | 117.63 | 84.27 | 71.07 | 66.04 |
| 54 | 220.00 | 111.62 | 80.20 | 67.57 | 62.80 |
| 55 | 207.00 | 105.54 | 76.04 | 64.01 | 59.49 |
| 56 | 194.00 | 99.44 | 71.86 | 60.43 | 56.17 |
| 57 | 182.00 | 93.79 | 67.99 | 57.09 | 53.07 |
| 58 | 170.00 | 88.08 | 64.04 | 53.72 | 49.94 |
| 59 | 158.00 | 82.31 | 60.03 | 50.28 | 46.75 |
| 60 | 146.00 | 76.49 | 55.95 | 46.81 | 43.53 |
| 61 | 135.00 | 71.13 | 52.19 | 43.61 | 40.55 |
| 62 | 123.00 | 65.17 | 47.97 | 40.02 | 37.21 |
| 63 | 112.00 | 59.69 | 44.07 | 36.72 | 34.15 |
| 64 | 101.00 | 54.15 | 40.10 | 33.36 | 31.02 |
| 65 | 90.00 | 48.54 | 36.05 | 29.95 | 29.95 |
| 66 | 80.00 | 43.42 | 32.35 | 26.83 | 24.96 |
| 67 | 69.00 | 37.69 | 28.16 | 23.33 | 21.70 |
| 68 | 59.00 | 32.45 | 24.33 | 24.33 | 24.33 |
| 69 | 49.00 | 27.12 | 20.40 | 16.85 | 15.67 |
| 70 | 39.00 | 21.75 | 16.41 | 13.54 | 12.59 |
| 71 | 29.00 | 16.29 | 12.33 | 10.16 | 9.44 |
| 72 | 19.00 | 10.75 | 8.16 | 8.16 | 8.16 |
| 73 | 9.00 | 5.13 | 3.91 | 3.21 | 2.99 |
